# The Smi-miR858a-*SmMYB* module regulates tanshinone and phenolic acid biosynthesis in *Salvia miltiorrhiza*

**DOI:** 10.1093/hr/uhae047

**Published:** 2024-02-23

**Authors:** Butuo Zhu, Meizhen Wang, Yongqi Pang, Xiangling Hu, Chao Sun, Hong Zhou, Yuxing Deng, Shanfa Lu

**Affiliations:** Key Lab of Chinese Medicine Resources Conservation, State Administration of Traditional Chinese Medicine of the People's Republic of China, Institute of Medicinal Plant Development, Chinese Academy of Medical Sciences & Peking Union Medical College, Beijing 100193, China; Engineering Research Center of Chinese Medicine Resource, Ministry of Education, Beijing 100193, China; Key Lab of Chinese Medicine Resources Conservation, State Administration of Traditional Chinese Medicine of the People's Republic of China, Institute of Medicinal Plant Development, Chinese Academy of Medical Sciences & Peking Union Medical College, Beijing 100193, China; Engineering Research Center of Chinese Medicine Resource, Ministry of Education, Beijing 100193, China; Key Lab of Chinese Medicine Resources Conservation, State Administration of Traditional Chinese Medicine of the People's Republic of China, Institute of Medicinal Plant Development, Chinese Academy of Medical Sciences & Peking Union Medical College, Beijing 100193, China; Engineering Research Center of Chinese Medicine Resource, Ministry of Education, Beijing 100193, China; Key Lab of Chinese Medicine Resources Conservation, State Administration of Traditional Chinese Medicine of the People's Republic of China, Institute of Medicinal Plant Development, Chinese Academy of Medical Sciences & Peking Union Medical College, Beijing 100193, China; Engineering Research Center of Chinese Medicine Resource, Ministry of Education, Beijing 100193, China; College of Pharmaceutical Sciences, Chengdu Medical College, Chengdu 610500, Sichuan, China; Key Lab of Chinese Medicine Resources Conservation, State Administration of Traditional Chinese Medicine of the People's Republic of China, Institute of Medicinal Plant Development, Chinese Academy of Medical Sciences & Peking Union Medical College, Beijing 100193, China; Engineering Research Center of Chinese Medicine Resource, Ministry of Education, Beijing 100193, China; Key Lab of Chinese Medicine Resources Conservation, State Administration of Traditional Chinese Medicine of the People's Republic of China, Institute of Medicinal Plant Development, Chinese Academy of Medical Sciences & Peking Union Medical College, Beijing 100193, China; Engineering Research Center of Chinese Medicine Resource, Ministry of Education, Beijing 100193, China; Key Lab of Chinese Medicine Resources Conservation, State Administration of Traditional Chinese Medicine of the People's Republic of China, Institute of Medicinal Plant Development, Chinese Academy of Medical Sciences & Peking Union Medical College, Beijing 100193, China; Engineering Research Center of Chinese Medicine Resource, Ministry of Education, Beijing 100193, China; Key Lab of Chinese Medicine Resources Conservation, State Administration of Traditional Chinese Medicine of the People's Republic of China, Institute of Medicinal Plant Development, Chinese Academy of Medical Sciences & Peking Union Medical College, Beijing 100193, China; Engineering Research Center of Chinese Medicine Resource, Ministry of Education, Beijing 100193, China

## Abstract

Tanshinones and phenolic acids are two major classes of bioactive compounds in *Salvia miltiorrhiza*. Revealing the regulatory mechanism of their biosynthesis is crucial for quality improvement of *S. miltiorrhiza* medicinal materials. Here we demonstrated that Smi-miR858a–Smi-miR858c, a miRNA family previously known to regulate flavonoid biosynthesis, also played critical regulatory roles in tanshinone and phenolic acid biosynthesis in *S. miltiorrhiza*. Overexpression of Smi-miR858a in *S. miltiorrhiza* plants caused significant growth retardation and tanshinone and phenolic acid reduction. Computational prediction and degradome and RNA-seq analyses revealed that Smi-miR858a could directly cleave the transcripts of *SmMYB6*, *SmMYB97*, *SmMYB111*, and *SmMYB112*. Yeast one-hybrid and transient transcriptional activity assays showed that Smi-miR858a-regulated SmMYBs, such as SmMYB6 and SmMYB112, could activate the expression of *SmPAL1* and *SmTAT1* involved in phenolic acid biosynthesis and *SmCPS1* and *SmKSL1* associated with tanshinone biosynthesis. In addition to directly activating the genes involved in bioactive compound biosynthesis pathways, SmMYB6, SmMYB97, and SmMYB112 could also activate *SmAOC2*, *SmAOS4*, and *SmJMT2* involved in the biosynthesis of methyl jasmonate, a significant elicitor of plant secondary metabolism. The results suggest the existence of dual signaling pathways for the regulation of Smi-miR858a in bioactive compound biosynthesis in *S. miltiorrhiza*.

## Introduction

*Salvia miltiorrhiza* Bunge (Danshen) is a well-known traditional Chinese medicine. Dried Danshen roots and rhizomes have been widely used for treating human diseases, such as Alzheimer’s disease, cerebrovascular and cardiovascular diseases, cancers, and so on [1, 2]. There are two major classes of bioactive compounds in Danshen, including the lipophilic tanshinones and the hydrophilic phenolic acids [2, 3]. Lipophilic tanshinones can be further classified into three groups, including diterpenoid tanshinones [e.g. tanshinone I (TAI), tanshinone IIA (TAII), cryptotanshinone (CT) and dihydrotanshinone I (DT-I)], tricyclic diterpenoid tanshinones (e.g. miltirone), and royleanone tanshinones (e.g. danshenxinkun A and isotanshinone I) [1], whereas hydrophilic phenolic acids mainly contain two groups, including hydroxybenzoic acids (e.g. *p*-hydroxybenzoic acid and gallic acid) and hydroxycinnamic acids [e.g. caffeic acid and rosmarinic acid (RA)] [1].

Biosynthesis of tanshinones starts from the mevalonate (MVA) pathway in the cytoplasm and the 2-C-methyl-d-erythritol 4-phosphate (MEP) pathway in the plastid. It leads to the production of the general units of isoprene, isopentenyl diphosphate (IPP), and dimethylallyl diphosphate (DMAPP) [4]. Then, geranylgeranyl diphosphate synthase (GGPPS), CPP synthase (CPS), kaurene synthase-like (KSL), cytochrome P450 mono-oxygenases (CYP76AH1, CYP76AH3, CYP76AK1, CYP71D375, CYP71D373), 2-oxoglutarate-dependent dioxygenase 3 (2OGD3), tanshinone IIA synthase (TIIAS), and other unknown enzymes are involved in catalyzing the subsequent reactions, resulting in the generation of final products [5–11]. Biosynthesis of phenolic acids starts from the tyrosine and phenylpropanoid pathways. Tyrosine aminotransferase (TAT), *p*-hydroxyphenylpyruvate reductase (HPPR), phenylalanine ammonia lyase (PAL), cinnamate 4-hydroxylase (C4H), 4-coumaroyl CoA ligase (4CL), rosmarinic acid synthase (RAS), and CYP98A14 are involved in the process. The regulation mechanism of these compounds has been intensively studied recently. Various transcription factor families are involved [1]. Among them, MYB is the largest family.

In *S. miltiorrhiza*, over 110 R2R3-MYBs have been identified. Several SmMYBs can regulate the biosynthesis of tanshinones or/and phenolic acids [1, 12]. For instance, SmMYB9b enhances tanshinone production through activating 1-deoxy-D-xylulose 5-phosphate synthase gene (*SmDXS2*), 1-deoxy-D-xylulose5-phosphate reductoisomerase gene (*SmDXR*) , *SmGGPPS*, and *SmKSL1* [13]. SmMYB39 inhibits the production of *p*-coumaric acid, RA, salvianolic acid B (Sal B), salvianolic acid A, and total phenolics through negatively regulating the expression of *SmC4H* and *SmTAT* [14]. SmMYB1 functions through positively regulating the expression of *SmTAT1*, *SmHPPR1*, *SmPAL1*, *SmC4H1*, *Sm4CL1*, *SmCYP98A14*, and *SmRAS1* [15]. SmMYB52 activates phenolic acid biosynthesis through regulating the expression of *SmTAT1*, *Sm4CL9*, *SmC4H1*, and *SmHPPR1* [16]. SmMYB111 stimulates phenolic acid biosynthesis through forming a regulatory complex with SmTTG1 and SmbHLH51 [17]. SmMYB97 activates tanshinone and phenolic acid biosynthesis through improving the expression of *SmCPS1*, *SmKSL1*, *SmPAL1*, and *SmTAT1* [18]. SmMYB98 activates the transcription of *SmGGPPS1*, *SmDXS2*, *SmKSL1*, *SmCYP76AH1*, *SmTAT1*, *SmPAL1*, *SmC4H1*, and *SmCYP98A14* to stimulate tanshinone and salvianolic acid biosynthesis [19]. SmMYB36 inhibits phenolic acid biosynthesis but promotes tanshinone accumulation in *S. miltiorrhiza* [20]. In addition, methyl jasmonate (MeJA) could also activate the biosynthesis of tanshinones and phenolic acids. For instance, external application of MeJA dramatically enhanced the accumulation of tanshinones and phenolic acids in *S. miltiorrhiza* hairy roots [21, 22]. Overexpression of MeJA biosynthesis genes, such as allene oxide cyclase gene (*SmAOC*) and jasmonic acid carboxyl methyltransferase gene (*SmJMT*) , could induce phenolic acid and tanshinone biosynthesis [23, 24].

miRNAs are a class of non-coding RNAs with size ~21 nt. They are generated from transcripts with internal stem–loop structures and play widespread biological roles in plant growth and development. miRNAs work mainly by targeting transcripts for cleavage [25]. miR858 is an miRNA conserved in plants, such as *Arabidopsis* [26], apple [27, 28], cotton [26], tomato [29], kiwifruit [30, 31], persimmon [32], grape [33], and potato [34]. Current knowledge suggests that miR858 is mainly involved in the regulation of flavonoid biosynthesis by cleaving transcripts encoding MYB transcription factors. For instance, persimmon miR858b inhibits proanthocyanidin accumulation by repressing the expression of *DkMYB19* and *DkMYB20* [32]. Apple mdm-miR858 regulates proanthocyanidin production by cleaving the transcripts of *MdMYB9/11/12* [28]. Kiwifruit miR858 regulates anthocyanin biosynthesis by repressing the expression of *AaMYBC1* [30]. *Arabidopsis* miR858 regulates flavonoid biosynthesis by cleaving *AtMYB11*, *AtMYB12*, and *AtMYB111* transcripts [35, 36].

Although the involvement of miR858 in regulating flavonoid biosynthesis has been revealed in several plants, its regulatory role for other secondary metabolites remains largely unknown. In this study, we identified *MIR858* in the *S. miltiorrhiza* genome and comprehensively analyzed its regulatory role in bioactive compound biosynthesis through a combination of computational prediction, genetic transformation, degradome analysis, RNA-sequencing (RNA-seq) analysis, ultra-high performance liquid chromatography (UPLC) detection, yeast one-hybrid assay (Y1H), and transient transcriptional activity assay (TTA). The results suggest that, in addition to flavonoid biosynthesis, *S. miltiorrhiza* miR858 also plays important roles in regulating phenolic acid and tanshinone biosynthesis through direct cleavage of *SmMYB6*, *SmMYB97*, *SmMYB111*, and *SmMYB112* transcripts. It subsequently affects the expression of genes involved in bioactive compound biosynthesis pathways. In addition, SmMYB6, SmMYB97, and SmMYB112 can activate *SmAOC2*, *SmAOS4*, and *SmJMT2* involved in the MeJA biosynthetic pathway, which may subsequently stimulate the biosynthesis of bioactive compounds. The results reveal a novel function of miR858 and provide important information for improving the quality of medicinal materials.

**Figure 1:**
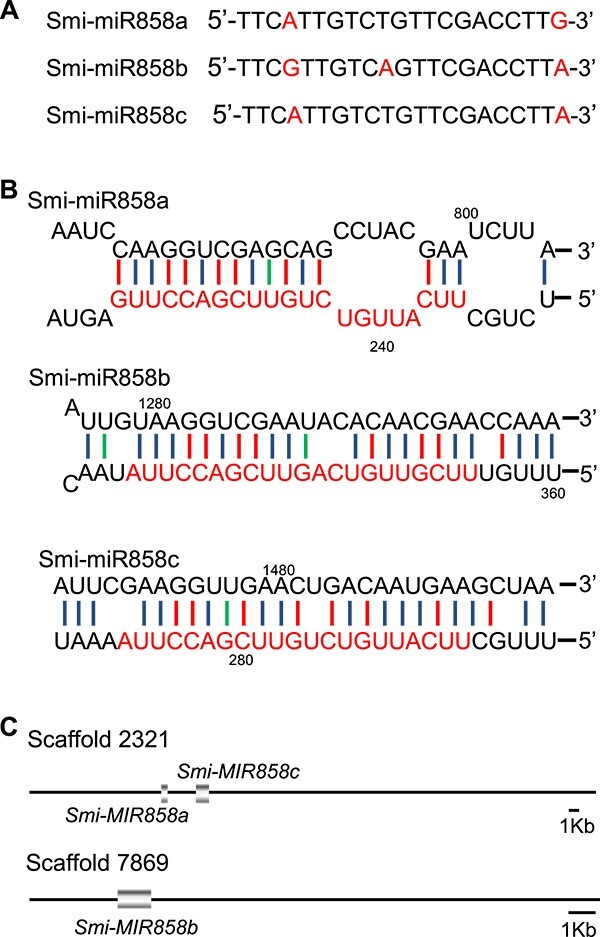
Analysis of Smi-miR858a/b/c in *S. miltiorrhiza*. **A** Sequences of Smi-miR858a, Smi-mi858b, and Smi-mi858c. Letters in red indicate different nucleotides among the three miRNAs. **B** Partial hairpin structures of *Smi-MIR858a*, *Smi-MIR858b*, and *Smi-MIR858c*. Smi-miR858a, Smi-miR858b, and Smi-miR858c sequences are indicated in red. Numbers indicate nucleotides numbered from the 5′ end in the corresponding precursors. **C** Schemes for the position of *Smi-MIR858a*, *Smi-MIR858b*, and *Smi-MIR858c* precursors in the scaffolds.

## Results

### Genome-wide identification of *MIR858* genes in *S. miltiorrhiza*

To identify all candidate *MIR858* genes in *S. miltiorrhiza*, we first carried out BLASTn analysis of known miR858 in miRBase (release 22.1) against the *S. miltiorrhiza* sRNA database [37–42]. This resulted in the identification of three mature Smi-miR858 sequences, termed Smi-miR858a, Smi-miR858b, and Smi-miR858c (Fig. 1A). Among them, Smi-miR858a and Smi-miR858c differ in the last nucleotide of the 3′ end, whereas Smi-miR858b has three and two nucleotide differences from Smi-miR858a and Smi-miR858c, respectively (Fig. 1A). We then mapped the three mature Smi-miR858 sequences to the genome of *S. miltiorrhiza* line 99-3 and predicted secondary structures for the genome sequence surrounding Smi-miR858s using RNA folding (Fig. 1B, Supplementary Data Figs S1–S3) [43, 44]. The predicted secondary structures were manually checked based on the criteria proposed by Meyers *et al*. [45]. As a result, a total of three *S. miltiorrhiza MIR858* precursors (*Smi-MIR858a*–*Smi-MIR858c*) were identified. Among them, *Smi-MIR858a* and *Smi-MIR858c* are located on scaffold 2321 with an interval of 2856 bp. *Smi-MIR858b* is located on scaffold 7869 (Fig. 1C). This suggests that the three *Smi-MIR858* genes identified by computational prediction are authentic and expressed. To further characterize the function of Smi-miR858, the expression level of Smi-miR858s was analyzed using sRNA data identified from five *S. miltiorrhiza* root small RNA libraries. As shown in Supplementary Data Fig. S4, Smi-miR858a was expressed at the highest levels in all five root tissues, Smi-miR858c was expressed at moderate levels, and Smi-miR858b was expressed at the lowest level.

### Smi-miR858s targeted R2R3-MYB transcription factor genes in *S. miltiorrhiza*

**Figure 2:**
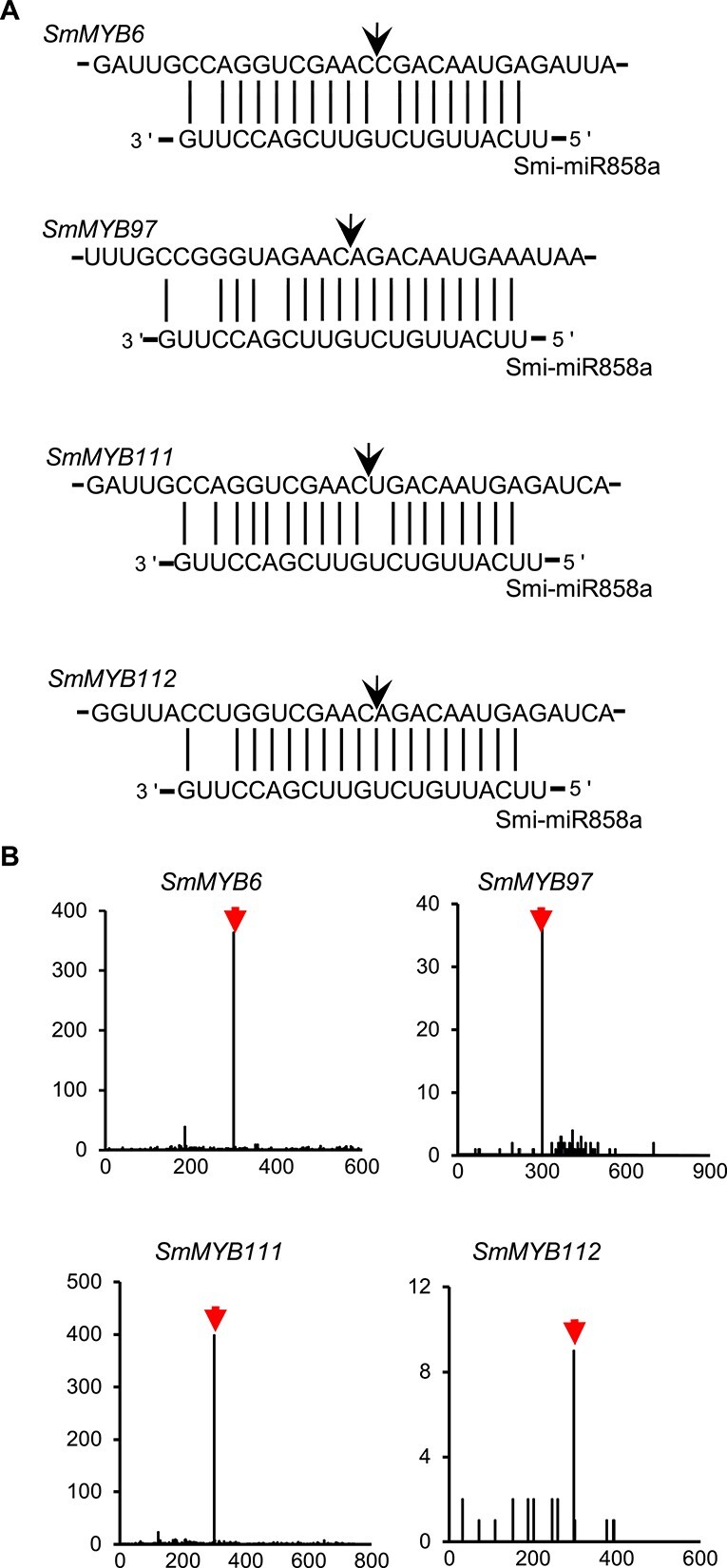
Analysis of Smi-miR858 targets. **A** Binding sites of Smi-miR858a in *SmMYB6*, *SmMYB97*, *SmMYB111*, and *SmMYB112*. Arrows indicate the 5′ termini of miRNA-guided cleavage products. **B** Degradome analysis of Smi-miR858a-mediated cleavage of *SmMYB6*, *SmMYB97*, *SmMYB111*, and *SmMYB112*. The *X*-axis shows the nucleotide position of the gene and the *Y*-axis shows the number of reads obtained by degradome sequencing. Black lines represent degradome fragments matched to the genes. Arrows indicate the products cleaved by Smi-miR858a.

Plant miRNAs can guide RNA-induced silencing complexes to target transcripts and then silence the targets mainly by direct cleavage at a site that corresponds to the 10th nucleotide from the miRNA 5′ end [25, 46]. The existence of perfect or near-perfect complementarity between miRNAs and their targets is critical in the process of target reorganization and subsequent cleavage. Thus, computational analysis of complementarity is an effective approach in predicting plant miRNA targets [47]. We first predicted the targets of Smi-miR858a on the online web servers TAPIR and psRNAtarget using the default parameters [48, 49]. This resulted in the prediction of a total of 11 targets (Fig. 2A, Supplementary Data Fig. S5, Supplementary Data Table S1), of which *SmMYB111* was targeted by three Smi-miR858s. *SmMYB6*, *SmMYB88*, *SmMYB97*, and *SMil_00003526* were targeted by Smi-miR858a and Smi-miR858c. The other six were targeted by either Smi-miR858a or Smi-miR858b (Supplementary Data Fig. S5, Supplementary Data Table S1). The Smi-miR858-complementary site of these *R2R3*-*SmMYB* transcripts was located in a region encoding the conserved imperfect repeat 3 (R3) [12].

Next, we aligned the degradome data of *S. miltiorrhiza* to the putative *SmMYB* targets. Significant accumulation of degraded fragments corresponding to the predicted cleavage sites were found for *SmMYB6*, *SmMYB97*, *SmMYB111*, and *SMil_00003526* (Fig. 2B). This suggests that the four *SmMYBs* are authentic targets of Smi-miR858s. Among them, SmMYB97 has been previously shown to promote tanshinone and phenolic acid accumulation by activating the promoters of *SmCPS1*, *SmKSL1*, *SmPAL1*, and *SmTAT1* [18]. SmMYB111 positively regulates phenolic acid biosynthesis by forming a transcription complex with SmTTG1 and SmbHLH51 [17]. SmMYB6 is a functionally unknown SmMYB identified in our previous studies [12]. SMil_00003526 is a novel SmMYB that has not been reported before. Here we named it SmMYB112. Phylogenetic analysis and multiple sequence alignment of SmMYB6, SmMYB97, SmMYB111, SmMYB112, and various R2R3-MYBs identified in *Arabidopsis thaliana*, *Zea mays*, *Malus domestica*, and *Vitis vinifera* showed that SmMYB6, SmMYB97, and SmMYB111 were R2R3-MYBs belonging to subgroup 7, whereas SmMYB112 was clustered with R2R3-MYBs in subgroup 6 (Supplementary Data Figs S6–S8, Supplementary Data Table S4). Except for *SmMYB6*, *SmMYB97*, *SmMYB111*, and *SmMYB112*, another seven predicted *SmMYB* targets could not be validated through degradome analysis. This indicates that these predicted targets could not be authentic targets of Smi-miR858s. The other possibility could be that the degradome data were not sufficient to validate these predicted targets.

### Overexpression of Smi-miR858a in *S. miltiorrhiza* plants repressed *SmMYB* targets and caused growth retardation

Since Smi-miR858a had the greatest number of putative targets among the identified three Smi-miR858s, could regulate the four validated *SmMYB* targets, and showed the highest expression in tissue analysis (Supplementary Data Figs S4 and S5, Supplementary Data Table S1), it was selected for transgenic analysis. To overexpress Smi-miR858a in *S. miltiorrhiza*, an artificial microRNA (amiRNA) vector, termed pHPT-858, was designed and constructed as described previously [50]. *Ptc-MIR408* precursor identified from *Populus trichocarpa* was used as the backbone [47]. Smi-miR858a and Smi-miR858a^*^ sequences were incorporated into the backbone to replace ptc-miR408 and ptc-miR408^*^ sequences, respectively. The constructed pHPT-858 was introduced into *S. miltiorrhiza* line 99-3 by *Agrobacterium tumefaciens*-mediated transformation as described before [51]. A total of 19 transgenic lines were obtained.

**Figure 3:**
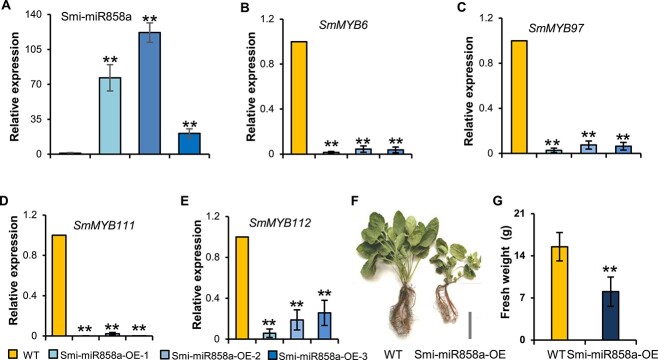
Analysis of Smi-miR858a overexpression in *S. miltiorrhiza*. **A** Relative expression level of Smi-miR858a in transgenic and wild-type lines. Bars represent standard deviations of the mean from three biological replicates (^**^P < 0.01, Student’s *t*-test). **B** Relative expression level of *SmMYB6* in transgenic and wild-type lines. **C** Relative expression level of *SmMYB97* in transgenic and wild-type lines. **D** Relative expression level of *SmMYB111* in transgenic and wild-type lines. **E** Relative expression level of *SmMYB112* in transgenic and wild-type lines. Bars are standard deviations of three biological replicates (^**^P < 0.01, Student’s *t*-test). **F** Phenotype of Smi-miR858a overexpression and wild-type line. Scale bar = 5 cm. **G** Comparison of fresh weight of wild-type and transgenic plants. Values are standard deviations determined by Student’s *t*-test (*n* = 15, ^**^P < 0.01).

Analysis of the Smi-miR858a level in transgenic lines using an miRNA-specific stem–loop RT–qPCR method [28] showed that the content of Smi-miR858a was increased dramatically (Fig. 3A). The expression level of *SmMYB* targets in transgenics and wild-type plants was comparatively analyzed using the RT–qPCR method. This showed that all four *SmMYB* genes were significantly downregulated in Smi-miR858a overexpression lines (Fig. 3B–E), and provides solid evidence for Smi-miR858a-mediated regulation of *SmMYB6*, *SmMYB97*, *SmMYB111*, and *SmMYB112* in *S. miltiorrhiza*. In accompaniment with the increase in Smi-miR858a and the downregulation of *SmMYB* targets, the transgenic plants showed significant growth retardation characterized by smaller leaves and roots and fewer leaves and roots (Fig. 3F). Fresh weight was decreased from 15.50 g in the wild type to 8.08 g in transgenic lines (Fig. 3G).

### Overexpression of Smi-miR858a significantly reduced tanshinone and phenolic acid production

**Figure 4:**
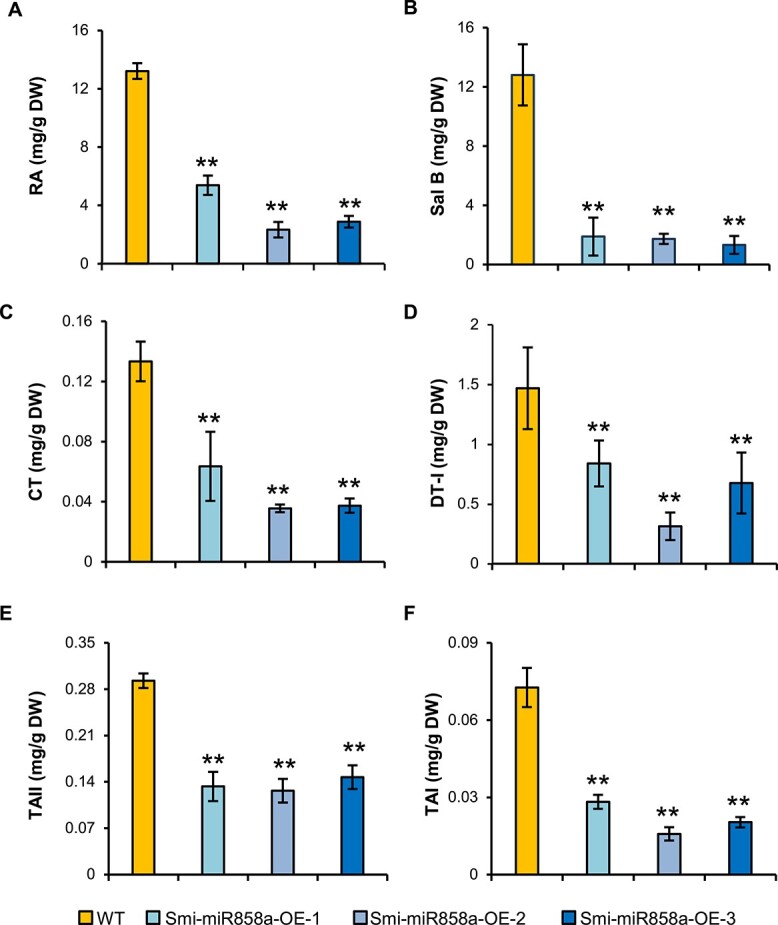
Analysis of phenolic acid and tanshinone contents in transgenic and wild-type lines. Contents of RA (**A**), Sal B (**B**), CT (**C**), DT-I (**D**), TAII (**E**) and TAI (**F**) in transgenic and wild-type roots. Bars represent standard deviations of the mean for three biological replicates determined by Student’s *t*-test (^**^P < 0.01).

It has been shown that miR858 regulates flavonoid biosynthesis in some plants [28, 30, 32, 35, 36]. However, there is no information about its regulatory role for other secondary metabolites. In order to gain insight into the precise role of Smi-miR858a in regulating secondary metabolism, we analyzed the content of phenolic acids and tanshinones in roots [1]. Dried roots of 3-month-old wild-type plants and artificial *Smi-MIR858a* transgenics were collected and analyzed using UPLC. The results showed that, compared with the wild-type plants, the average contents of RA and Sal B in Smi-miR858a transgenics were reduced by 73.2 and 87.2%, respectively (Fig. 4A and B). Analysis of the four major tanshinones, CT, DT-I, TAII, and TAI, showed that their average contents in roots of *Smi-MIR858a* transgenics were decreased by 65.9, 58.5, 53.7, and 70.4%, respectively (Fig. 4C–F). The results indicate that Smi-miR858a negatively regulates phenolic acid and tanshinone biosynthesis in *S. miltiorrhiza*.

### Effects of Smi-miR858a on global gene expression

**Figure 5:**
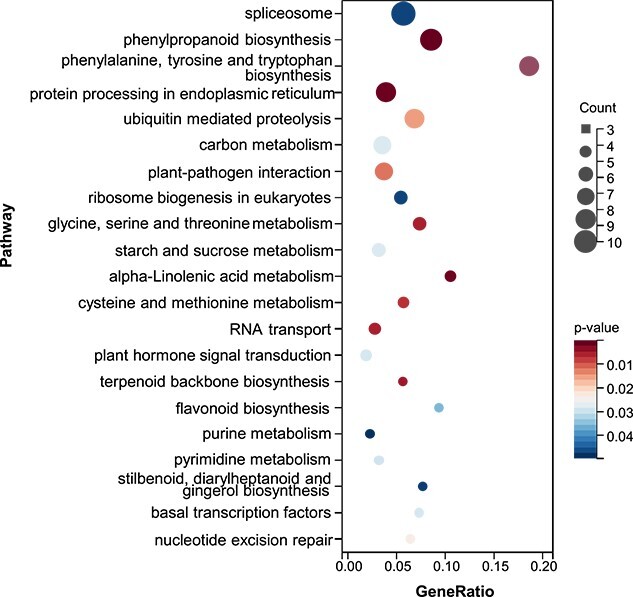
KEGG analysis of DEGs in transgenic and wild-type lines*.* The top 21 most statistically significant terms of KEGG pathways. The *X*-axis represents GeneRatio and the *Y*-axis represents KEGG pathways. The size of circle represents gene number. Different color of circles represents adjusted *P* value.

To further clarify the biological role of miR858 in *S. miltiorrhiza*, RNA-seq analysis was performed to detect differentially expressed genes (DEGs) between wild-type and miR858 overexpression lines. The analysis was carried out for roots from a 3-month-old wild-type line and three transgenic lines. Three replicates for each line were performed. The average value of FPKM (fragments per kilobase of transcript per million fragments mapped) was used for comparison. Significantly up- and downregulated genes (|log2(foldchange)| > 2 and *P* < 0.05) were screened. This resulted in the identification of 845 DEGs, of which 305 were upregulated and 540 downregulated (Supplementary Data Table S3). KEGG analysis showed that DEGs involved in ‘phenylpropanoid biosynthesis’, ‘phenylalanine, tyrosine and tryptophan biosynthesis’, ‘alpha-linolenic acid metabolism’, ‘terpenoid backbone biosynthesis’, ‘flavonoid biosynthesis’, and ‘stilbenoid, diarylheptanoid and gingerol biosynthesis’ were significantly enriched (Fig. 5).

The regulatory role of miR858 in flavonoid biosynthesis has been previously found in various plants, such as *Arabidopsis*, persimmon, apple, and kiwifruit [28, 30, 32, 35, 36]. The enrichment of DEGs in the flavonoid biosynthesis pathway suggests that Smi-miR858a plays a conserved function in regulating flavonoid biosynthesis in *S. miltiorrhiza*. The enrichment of DEGs in the major bioactive compound biosynthesis pathways, such as the terpenoid backbone biosynthesis pathway for tanshinone production and the phenylpropanoid biosynthesis pathway related to phenolic acid production, is consistent with the results from phenolic acid and tanshinone content determination (Fig. 4). In addition, the α-linolenic acid metabolism pathway is associated with the production of MeJA [21, 24]. The enrichment of DEGs in this pathway provides another layer of evidence for elucidating the role of Smi-miR858 in regulating bioactive compound biosynthesis.

### Overexpression of Smi-miR858a altered the expression of genes in tanshinone, phenolic acid, and flavonoid biosynthesis pathways

**Figure 6:**
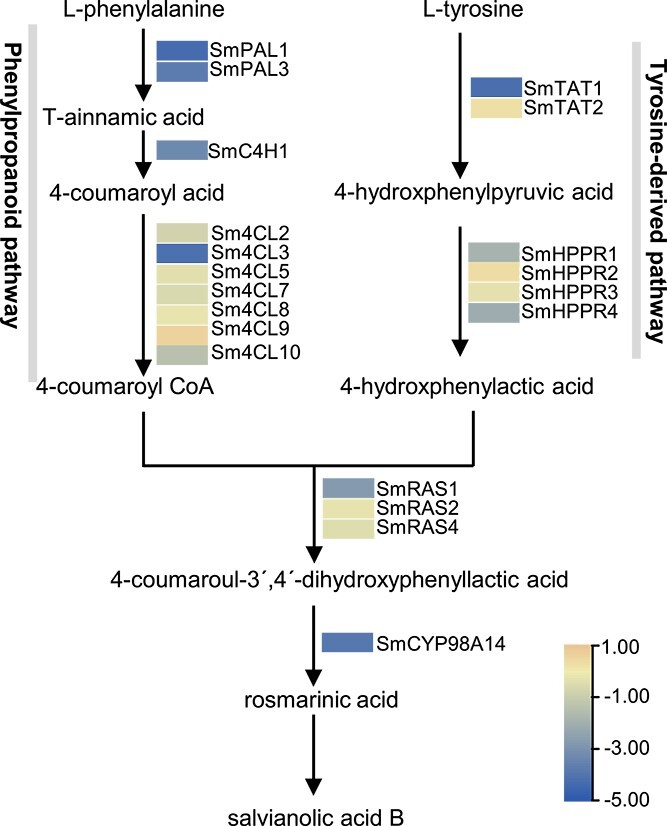
RNA-seq analysis of genes involved in the phenolic acid biosynthesis pathway in transgenic and wild-type lines. The average FPKM of three biological replicates was used for comparison.

Since bioactive compound contents were reduced in *Smi-miR858a* overexpression plants and DEGs were enriched in the biosynthetic pathways (Figs 4 and 5), we analyzed the expression level of genes related to the biosynthetic pathways. The results showed that, in comparison with wild-type plants, 20 phenolic acid biosynthesis-related genes were differentially expressed. Among them, eight genes, *SmPAL1*, *SmPAL3*, *SmC4H1*, *Sm4CL3*, *SmTAT1, SmRAS1*, *SmHPPR4* and *SmCYP98A14*, were significantly downregulated (|log2(foldchange)| > 2). Nine genes, *Sm4CL2*, *Sm4CL5*, *Sm4CL7*, *Sm4CL8*, *Sm4CL10*, *SmHPPR1*, *SmHPPR3*, *SmRAS2*, and *SmRAS4*, were slightly downregulated (|log2(foldchange)| ≤ 2). The other three genes, *Sm4CL9*, *SmTAT2*, and *SmHPPR2*, were slightly upregulated (|log2(foldchange)| ≤ 2) (Fig. 6).

**Figure 7:**
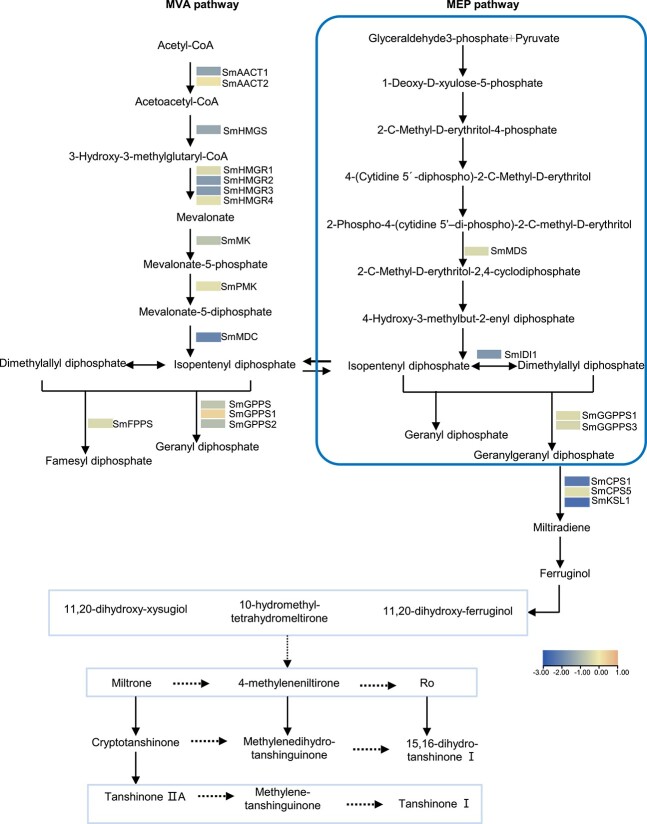
RNA-seq analysis of genes involved in tanshinone biosynthesis pathway in transgenic and wild-type lines. The average FPKM of three biological replicates was used for comparison.

Analysis of the genes related to tanshinone biosynthesis identified 21 DEGs. Among them, three genes, *SmMDC*, *SmCPS1*, and *SmKSL1* (|log2(foldchange)| > 2), were significantly downregulated. Sixteen genes, *SmAACT1*, *SmHMGS*, *SmHMGR1*–*SmHMGR4*, *SmMK*, *SmPMK*, *SmFPPS*, *SmGPPS*, *SmGPPS2*, *SmGGPPS1*, *SmGGPPS3*, *SmIDI1*, *SmMDS*, and *SmCPS5*, were slightly downregulated (|log2(foldchange)| ≤ 2). The other two, *SmAACT2* and *SmGPPS1*, were slightly upregulated (|log2(foldchange)| ≤ 2) (Fig. 7).

In addition, 17 genes involved in flavonoid biosynthesis were differentially expressed. These include 3 that were significantly downregulated (anthocyanidin synthase gene (*SmANS*), chalcone isomerase gene (*SmCHS1*), and flavonoid 3′-hydroxylase gene (*SmF3′H2*) ) (|log2(foldchange)| > 2), 10 that were slightly downregulated (*SmF3H1*, *SmF3′H4*, *SmF3′H5*, *SmF3′H6*, flavonol synthase gene (*SmFLS2*) , *SmCHI1*, *SmCHI2*, *SmCHS1*, chalcone synthase gene (*SmCHS2*) , and *SmCHS7*) (|log2(foldchange)| ≤ 2), and 4 that were upregulated (*SmF3′H3*, *SmCHI3*, *SmCHS3*, and *SmCHS6*) (Supplementary Data Fig. S9). The results further suggest the involvement of miR858 in regulating flavonoid biosynthesis [28, 30, 32, 35, 36].

### Smi-miR858a targeted *SmMYB*s to regulate the expression of downstream genes

Through computational prediction and degradome analysis, we found that *SmMYB6*, *SmMYB97*, *SmMYB111*, and *SmMYB112* were the targets of Smi-miR858s (Fig. 2). Consistently, overexpression of Smi-miR858a caused significant downregulation of the four *SmMYBs* (Fig. 3). Among them, SmMYB97 activates tanshinone and phenolic acid biosynthesis [18]. SmMYB111 is a member of the transcription complex SmTTG1–SmMYB111–SmbHLH51. It positively regulates the expression of genes involved in phenolic acid biosynthesis [17]. The functions of SmMYB6 and SmMYB112 remain to be elucidated.

**Figure 8:**
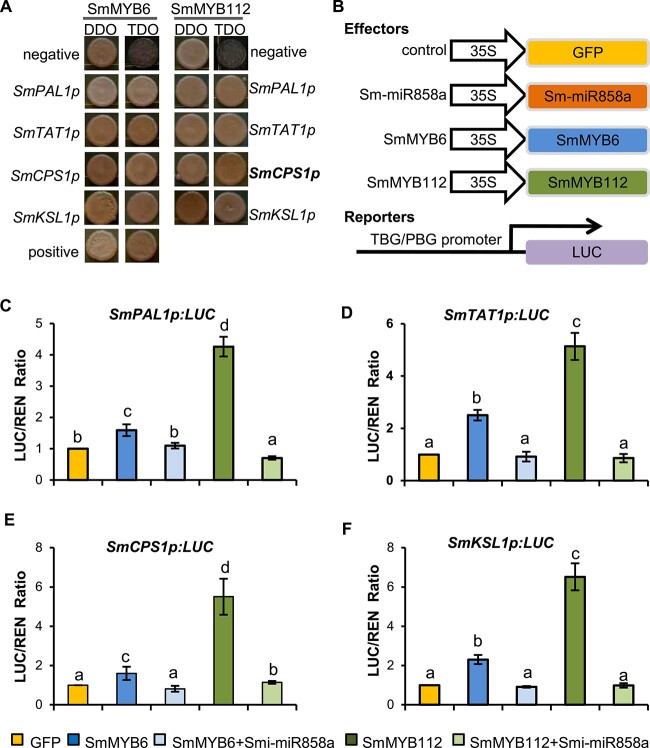
Binding of SmMYB6 and SmMYB112 to tanshinone and phenolic acid biosynthesis-related genes. **A** Binding of SmMYB6 and SmMYB112 to *SmPAL1*, *SmTAT1*, *SmCPS1*, and *SmKSL1* promoters in yeasts. p53HIS2/pGADT7-p53 vectors were used as positive control. p53HIS2/pGADT7-SmMYB6 and p53HIS2/pGADT7-SmMYB112 were used as negative controls. **B** Schemes of the reporter and effector constructs used in transient transcriptional activity. TBG, tanshinone biosynthesis genes. PBG, phenolic acid biosynthesis genes. **C**–**F** Transcriptional activity of SmMYB6 and SmMYB112 on the promoters of *SmPAL1*, *SmTAT1*, *SmCPS1*, and *SmKSL1* in tobacco leaves. Bars are standard deviations of three biological replicates. Lowercase letters indicate a significant difference determined by one-way ANOVA and *post hoc* Tukey’s test (*P* = 0.05).

In order to identify the role of SmMYB6 and SmMYB112 in tanshinone and phenolic acid biosynthesis, yeast one-hybrid (Y1H) assays were performed as reported previously [18]. Since the expression of *SmKSL1*, *SmCPS1*, *SmPAL1*, and *SmTAT1* was significantly repressed in Smi-miR858a overexpression plants (Figs 6 and 7), the promoter regions of these genes were selected for further analysis. To construct Y1H vectors, the MYB-binding site-containing promoter regions of the *SmKSL1*, *SmCPS1*, *SmPAL1*, and *SmTAT1* genes (*SmKSL1p*, *SmCPS1p*, *SmPAL1p*, and *SmTAT1p*) were cloned into the pHIS2 vector. The CDSs of *SmMYB6* and *SmMYB112* were cloned into pGADT7-Rec2. Constructs with promoter regions and *SmMYB* coding regions were co-transformed into yeasts. The pairs, comprising p53HIS2/pGADT7-p53, p53HIS2/pGADT7-SmMYB6, and p53HIS2/pGADT7-SmMYB112, were used as positive and negative controls, respectively. The results showed that yeast cells co-transformed with *SmPAL1p*/*SmMYB6*, *SmPAL1p*/*SmMYB112*, *SmTAT1p*/*SmMYB6*, *SmTAT1p*/*SmMYB112*, *SmCPS1p*/*SmMYB6*, *SmCPS1p*/*SmMYB112*, *SmKSL1p*/*SmMYB6*, or *SmKSL1p*/*SmMYB112* could grow on both SD/−Leu/−Trp medium (DDO) and SD/−Leu/ −Trp/−His (TDO) media as the positive control, whereas the cells with negative control could grow only on DDO medium (Fig. 8A). This suggests that SmMYB6 and SmMYB112 could bind to the promoters of *SmCPS1 SmKSL1*, *SmPAL1*, and *SmTAT1*.

Next, we asked whether SmMYB6 and SmMYB112 could activate the transcription of *SmCPS1 SmKSL1*, *SmPAL1*, and *SmTAT1*. In order to address this question, a transient transcriptional activity [29] assay was conducted. The promoter regions of *SmCPS1 SmKSL1*, *SmPAL1*, and *SmTAT1* used for Y1H were fused with the LUC reporter gene. The vectors were then co-transformed into tobacco leaves with *35S:GFP*, *35S:Smi-miR858a*, *35S:SmMYB6*, or *35S:SmMYB112*. Analysis of the luminescence intensity showed that SmMYB6 and SmMYB112 could significantly increase the luminescence intensity (Fig. 8B–F). This indicates that both SmMYB6 and SmMYB112 can activate the expression of *SmKSL1*, *SmCPS1*, *SmPAL1*, and *SmTAT1*. In addition, co-transformation of *35S:Smi-MIR858a* and *35S:SmMYB6* or *35S:Smi-MIR858a* and *35S:SmMYB112* with the reporters into tobacco leaves resulted in significant reduction of fluorescence intensity in comparison with those without 35S:Smi-miR858a. It further confirms that Smi-miR858a negatively regulated the expression of *SmMYB6* and *SmMYB112*.

### Smi-miR858a targeted *SmMYBs* to regulate methyl jasmonate biosynthesis genes

**Figure 9:**
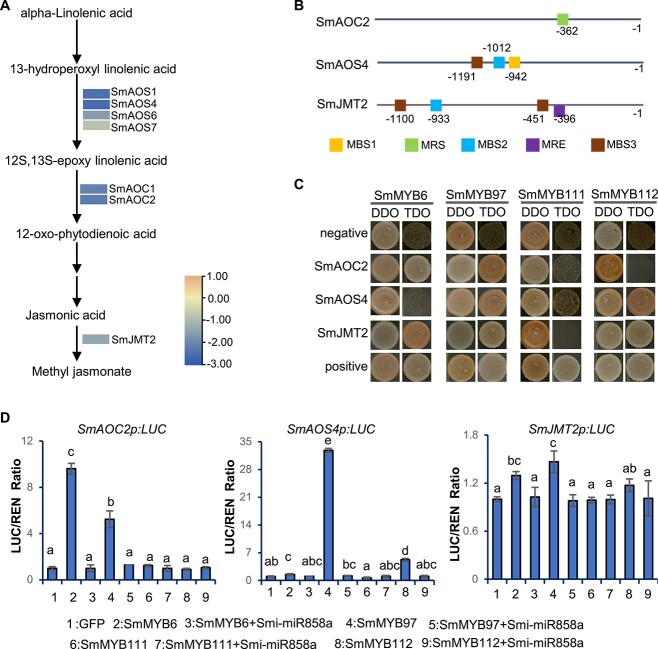
Binding of SmMYBs to MeJA biosynthesis-related genes. **A** RNA-seq analysis of genes involved in MeJA biosynthesis pathway in transgenic and wild-type lines. The average FPKM of three biological replicates was used for comparison. **B** Distribution of MYB-binding sites in *SmAOC2*, *SmAOS4*, and *SmJMT2* promoters. MBS1, CAACTG; MRS, CCGTTG; MBS2, CAACCA; MBS3, TAACCA; MRE, AACCTAA. **C** Binding analysis of SmMYB6, SmMYB97, SmMYB111, and SmMYB112 to *SmAOC2*, *SmAOS4*, and *SmJMT2* promoters in yeasts. p53HIS2 and pGADT7-p53 were used as positive controls. p53HIS2 and pGADT7-SmMYBs were used as negative controls. **D** Transcriptional activity of SmMYB6, SmMYB97, SmMYB111, and SmMYB112 on the promoters of *SmAOC2*, *SmAOS4*, and *SmJMT2* in tobacco leaves. Bars are standard deviations of three biological replicates. Lowercase letters indicate significant difference determined by one-way ANOVA and *post hoc* Tukey’s test (*P* = 0.05).

KEGG analysis showed that DEGs involved in the α-linolenic acid metabolism pathway were enriched (Fig. 5). Further analysis of genes involved in the pathway showed that *SmAOS1*, *SmAOS4*, *SmAOS6*, *SmAOS7*, *SmAOC1*, *SmAOC2*, and *SmJMT2* were downregulated in Smi-miR858a overexpression plants in comparison with their expression in wild-type plants (Fig. 9A). To reveal the regulatory role of SmMYB6/97/111/112 in MeJA biosynthesis, Y1H and TTA experiments were performed. Since the expression of *SmAOC2*, *SmAOS4*, and *SmJMT2* showed the most significant downregulation, their promoter regions were selected for further analysis. As shown in Fig. 9B, all three promoters contain the MYB-responsive elements.

For Y1H, promoter regions of *SmAOC2*, *SmAOS4*, and *SmJMT2* (*SmAOC2p*, *SmAOS4p*, and *SmJMT2p*) were cloned into the pHIS2 vector. The CDSs of *SmMYB6*, *SmMYB97*, *SmMYB111*, and *SmMYB112* were cloned into pGADT7-Rec2 as previously described. Constructs with promoter regions and *SmMYB* coding regions were co-transformed into yeasts and then analyzed for growth. The p53HIS2/pGADT7-p53 construct pair was used as a positive control. The construct pairs p53HIS2/pGADT7-SmMYB6, p53HIS2/pGADT7-SmMYB97, p53HIS2/pGADT7-SmMYB111 and p53HIS2/pGADT7-SmMYB112 were used as negative controls. As shown in Fig. 9C, yeast cells co-transformed with *SmAOC2p*/ *SmMYB6*, *SmJMT2p*/*SmMYB6*, *SmAOC2p*/*SmMYB97*, *SmAOS4p*/ *SmMYB97*, *SmJMT2p*/*SmMYB97*, *SmAOS4p*/*SmMYB112*, and *SmJMT2p*/*SmMYB112* could grow on DDO and TDO media as the positive control. The results suggests that SmMYB6 could bind to the promoters of *SmAOC2* and *SmJMT2*. SmMYB97 could bind to the promoters of *SmAOC2*, *SmAOS4*, and *SmJMT2.* SmMYB112 could bind to the promoters of *SmAOS4* and *SmJMT2* (Fig. 9C).

For the TTA assay, the same promoter regions of *SmAOC2*, *SmAOS4*, and *SmJMT2* used for Y1H were fused with the reporter gene and co-transformed into tobacco leaves with *35S:GFP*, *35S:SmMYB6*, *35S:SmMYB97*, *35S:SmMYB111*, *35S:SmMYB112*, or *35S:Smi-miR858a*. Luminescence intensity measurement showed that the expression of *SmAOC2* could be significantly activated by SmMYB6 and SmMYB97. The expression of *SmAOS4* could be significantly activated by SmMYB97 and SmMYB112. The expression of *SmJMT2* could be activated by SmMYB6 and SmMYB97, although the degree of activation was relatively low in comparison with the activation of SmMYB6 and SmMYB97 on *SmAOC2* and SmMYB97 and SmMYB112 on *SmAOS4* (Fig. 9D)*.* This also showed that the activation activities were inhibited by Smi-miR858a. The results are consistent with those from Y1H (Fig. 9C), suggesting regulatory role of Smi-miR858a in MeJA biosynthesis through targeting *SmMYB* transcripts.

## Discussion

### MiR858 is a key regulator of bioactive compound biosynthesis in plants

MYBs, characterized by the deeply conserved MYB domain, are composed of the largest transcription factor family in plants. Members of the family play significant regulatory roles in the biosynthesis of bioactive compounds [1, 25]. Several miRNAs, including miR159, miR319, miR828, and miR858, are involved in the post-transcriptional regulation of plant *MYB* genes [12]. Among them, miR858 has the greatest number of *MYB* targets, since its target site is located in the deeply conserved 3′ end of the R3 repeats of *MYB* genes [12]. Previous studies have shown that several miR858-targeted *MYB* genes are involved in flavonoid biosynthesis [28, 30, 32, 35, 36]. Through cleaving the transcripts of these *MYB* genes, miR858 could regulate the biosynthesis of flavonoids and their derivates, such as anthocyanins and proanthocyanidins, in *Arabidopsis*, persimmon, apple, and kiwifruit [28, 30, 32, 35, 36].

*Salvia miltiorrhiza* Bunge is a perennial plant in the *Salvia* genus of the Lamiaceae family [1]. The main bioactive compounds, phenolic acids, and tanshinones, have been clinically used for management of vascular diseases and various other diseases [2, 3]. In addition, *S. miltiorrhiza* also produces other bioactive compounds, such as anthocyanidins, flavonoids, monoterpenes, sesquiterpenes, proanthocyanidins, triterpenes, and so on [1]. Overexpression of Smi-miR858a caused significant downregulation of flavonoid biosynthesis enzyme genes, such as *SmANS*, *SmCHI1*, and *SmF3′H2*. This suggests that the regulatory role of miR858 in flavonoid biosynthesis is conserved among *S. miltiorrhiza* and other eudicotyledon species. In addition to flavonoids, overexpression of Smi-miR858a also caused significant decreases in tanshinone and phenolic acid contents. The results reveal novel functions of miR858 and suggest that Smi-miR858a is a negative regulator of phenolic acid, tanshinones, and flavonoids in *S. miltiorrhiza*. Considering the significance of MYBs in secondary metabolism and the conservation of miR858 in plants, it is very likely that the regulatory role of miR858 in the biosynthesis of phenolic acids and terpenoids in *S. miltiorrhiza* is also conserved in other plant species, although this hypothesis remains to be tested. In addition, Smi-miR858a-overexpressing transgenic plants showed significant growth retardation characterized by smaller leaves and roots and fewer leaves and roots (Fig. 3F). This indicates that Smi-miR858a also plays important roles in plant growth and development.

### MiR858 regulated bioactive compound biosynthesis through dual signaling pathways

Through computational analysis, we predicted a total of 11 *SmMYBs* to be targets of three Smi-miR858s. Among them, *SmMYB6*, *SmMYB97*, *SmMYB111*, and *SmMYB112* were validated through degradome analysis and Smi-miR858a overexpression. Previous studies suggest that *SmMYB97* acts as a positive regulator of tanshinone and phenolic acid biosynthesis [18]. SmMYB111 activates phenolic acid biosynthesis through forming a transcription complex with SmbHLH51 and SmTTG1 [17]. Here, we analyzed the function of SmMYB6 and SmMYB112 using Y1H and TTA assays. We found that both of them could activate the expression of *SmKSL1*, *SmCPS1*, *SmPAL1*, and *SmTAT1*, of which *SmKSL1* and *SmCPS1* are involved in tanshinone biosynthesis, whereas *SmPAL1* and *SmTAT1* are involved in phenolic biosynthesis. Since overexpression of Smi-miR858a significantly not only downregulated the four enzyme genes mentioned above but also various other genes related to flavonoid tanshinone and phenolic acid biosynthesis (Figs 6 and 7, Supplementary Data Fig, S9), we could not exclude the possibility that some of these enzyme genes were also regulated by the *SmMYB* targets of Smi-miR858a.

In addition to binding the promoters of bioactive compound biosynthesis pathway genes, SmMYB6, SmMYB97, and SmMYB112 could also activate genes involved in the MeJA biosynthesis pathway through binding to the promoters of these genes (Fig. 9). Among them, SmMYB6 could activate the expression of *SmAOC2* and *SmJMT2*. SmMYB97 could activate the expression of *SmAOC2*, *SmAOS4*, and *SmJMT2.* SmMYB112 could activate the expression of *SmAOS4*. Since overexpression of MeJA biosynthesis-related genes and external application of MeJA could significantly enhance phenolic acid and tanshinone production in *S. miltiorrhiza* [21–24], the upregulation of *SmAOC2*, *SmAOS4*, and *SmJMT2* by Smi-miR858a-targeted *SmMYB6*, *SmMYB97*, and *SmMYB112* suggests another layer of the signaling pathway for the regulation of Smi-miR858a in bioactive compound biosynthesis.

### Significance of miRNA-mediated post-transcriptional regulation in bioactive compound biosynthesis in *S. miltiorrhiza*

So far, hundreds of miRNAs have been identified in *S. miltiorrhiza* [40–42, 52–56]. Many of them have been implicated in the regulation of bioactive compound biosynthesis. For instance, *S. miltiorrhiza* miR156a and miR156b targets eight SQUAMOSA promoter binding protein‐like genes (*SmSPLs*) that could be associated with anthocyanin biosynthesis [4, 57]. Smi-miR12112 could be a regulator of phenolic acid metabolism through targeting polyphenol oxidase (*SmPPO*) genes for cleavage [52, 55]. Smi-miR5072 may participate in terpenoid backbone biosynthesis through regulating the enzyme gene acetyl-CoA C-acetyltransferase gene (*SmAACT*) [40]. Smi-miR7972 plays a role in tanshinone biosynthesis through regulating DEMETER-like DNA glycosylase 1 (*SmDML1*), a gene associated with DNA methylation and demethylation in *S. miltiorrhiza* [54, 58]. Smi-miR319, Smi-miR159, and Smi-miR828 regulate anthocyanin and phenolic acid biosynthesis through targeting a subset of *SmMYB* genes [12]. The results indicate the significance of miRNAs in the biosynthesis of secondary metabolites in *S. miltiorrhiza*, although the function of these miRNAs remains to be validated through plant genetic transformation.

Recently, Zheng *et al*. overexpressed Smi-miR396b in hairy roots of *S. miltiorrhiza* [59]. They found that Smi-miR396b downregulated salvianolic acid biosynthesis and upregulated tanshinone production through targeting growth-regulating factor genes (*SmGRFs*), histone deacetylase gene (*SmHDT1*) , and *SmMYB37/4* genes. In addition, Zou *et al*. constructed an amiRNA-mediated miR408-suppressed expression vector to inhibit the expression of Smi-miR408 in *S. miltiorrhiza* plants [60]. The results showed that the content of phenolic acids increased in the transgenic lines. Further analysis showed that the regulatory role of Smi-miR408 in phenolic acid biosynthesis was mediated by targeting the transcripts of laccase gene (*SmLAC3*) gene for cleavage [60]. In this study, we found that Smi-miR858a played significant regulatory role in the biosynthesis of phenolic acids, tanshinones, and flavonoids through targeting a subset of *SmMYB* genes. This further confirms the significance of miRNA-mediated post-transcriptional regulation in bioactive compound production in *S. miltiorrhiza*.

Because of their vital regulatory role in secondary metabolite biosynthesis, miRNAs of medicinal plants have become a bright research field [25]. But information on the function and regulatory network of medicinal plant miRNAs is very limited. Greater efforts are needed to authenticate medicinal plant miRNA candidates and uncover their functions through genetic transformation. From this point of view, elucidating the regulatory role of Smi-miR858 in bioactive compounds is meaningful.

## Materials and methods

### Plant materials and growth conditions

Sterile plantlets of *S. miltiorrhiza* (line 99-3) were grown on 1/2MS agar medium at 25°C for 40 days under a photoperiod of 16 h light/8 h dark. Leaf discs from the sterile plantlets were used for *Agrobacterium*-mediated transformation as described previously [51]. For transient transcriptional activity assay [29], *Nicotiana benthamiana* seeds were sown in soil and grown at 25°C for 1 month with the photoperiod of 16 h light/8 h dark in a greenhouse.

### *Smi-MIR858* gene identification and RNA secondary structure prediction

Plant miR858 sequences in miRBase (release 22.1) were used for BLASTn analysis against the *S. miltiorrhiza* small RNA database [37, 38, 40–42, 56]. The identified Smi-miR858s were then used for BLASTn analysis against the whole genome of *S. miltiorrhiza* line 99-3 [38, 43]. Secondary structures of the possible Smi-miR858 sequences were predicted using mFold on the RNAfold web server (http://www.unafold.org/RNA_form.php). Manual examination of the predicted secondary structures was carried out based on the criteria proposed previously [45]. The existence of identified *Smi-MIR858*s was confirmed by BLASTn analysis of *Smi-MIR858*s against the NCBI EST databases and the RNA-seq reads of *S. miltiorrhiza* line 99-3 (https://www.ncbi.nlm.nih.gov/sra).

### Smi-miR858 target gene prediction

The complementarity of *SmMYB*s to the mature Smi-miR858 sequences was analyzed using psRNATarget and the TAPIR web (http://bioinformatics.psb.ugent.be/webtools/tapir/) with the default parameters [48, 49].

### Plasmid construction and plant transformation

Artificial microRNA 858a (amiR858a) was designed as previously reported [50]. The *pBI-MIR408* binary vector was used as the template. Three pairs of primers were designed (Supplementary Data Table S2) and used in different combinations to conduct PCR amplification. The three prime combinations were 35sP/4P, 3P/2P, and 1P/NosP, respectively. The amplified three fragments contained overlapping sequences of Smi-miR858a and Smi-miR858a^*^. Overlapping PCR using the 35SP/NosP primer set generated a new fragment containing the Smi-miR858a and Smi-miR858a^*^ sequences. The fragment was digested with SacI and BamHI, and then cloned into pMD18-T vector. After sequence verification, the fragment was inserted into pGPTV-HPT to generate the pHPT-858 overexpression vector. The vector was transformed into *A. tumefaciens* GV3101. *Agrobacterium*-mediated transformation of *S. miltiorrhiza* was conducted as previously described [51].

### Gene expression analysis

*Salvia miltiorrhiza* tissues were used for total RNA extraction using the Plant Total RNA Extraction kit (Aidlab, China) according to the user manual. Total RNA was transcribed into cDNA using the TransScript-Uni One-Step gDNA Removal and cDNA Synthesis SuperMix kit (TransGen Biotech). Reverse transcription of Smi-miR858 was carried out with specific stem-loop primers. RT–qPCR was performed using the TransStart Tip Green qPCR SuperMix (TransGen Biotech) and analyzed using the Bio-Rad CFX96 detection system. Three biological replicates and four technical replicates for each biological replicate were performed. *S. miltiorrhiza* 5.8S rRNA and *SmUbiquitin* were used as internal controls for the analysis of miR858s and protein-coding genes, respectively. The primers used for RT–PCR analysis are listed in Supplementary Data Table S2.

### Degradome library construction and sequencing

For the construction of degradome library, total RNAs from six different tissues, (young root, mature root, stem, leaf, juvenile flower, and mature flower) were pooled. The library was constructed as previously described [61]. Polyadenylated RNA molecules were isolated using oligo(dT), and then single-stranded 5′ RNA adapters were ligated to the mRNA fragments using T4 RNA ligase (Ambion). Then, the RNA fragments were reverse-transcribed into cDNA using Superscript III Reverse Transcriptase (Invitrogen). After digestion with MmeI, the 3′ DNA adapter was ligated to the DNA fragments. Subsequently, PCR were performed and the products were sequenced.

### Phylogenetic analysis

Alignment of amino acid sequences of SmMYB6, SmMYB97, SmMYB111, SmMYB112, and *A. thaliana*, *Z. mays*, *M. domestica*, and *V. vinifera* R2R3 MYBs in subgroups 5, 6, and 7 and construction of neighbor-joining phylogenetic trees were carried out using MEGA6 software as previously described [62].

### RNA sequencing and gene expression analysis

Total RNA from the roots of wild-type and *Smi-MIR858a* overexpression lines was used for RNA-seq [63]. After mRNA isolation, fragmentation, cDNA synthesis and purification, cDNA libraries were generated and sequenced using the Illumina HiSeq platform. Clean reads were obtained and then mapped to the draft genome of *S. miltiorrhiza* line 99-3 using HISAT2. Gene expression levels were estimated by FPKM. The average FPKM values of wild-type and *Smi-MIR858a* overexpression lines were used for comparison. Genes with significantly different expression (|log2(foldchange)| > 2, *P* < 0.05) were considered to be DEGs (Supplementary Data Table S3).

### UPLC analysis of phenolic acids and tanshinones

Fresh roots of 3-month wild type and *Smi-MIR858a*-overexpressed transgenic plants were collected. They were dried at 30°C. The contents of tanshinones were extracted and detected as previously described [9]. For phenolic acid detection, 1 ml of 80% methanol was added to 0.1 g of powder, and then sonicated for 20 min, followed by centrifugation at 8000 rpm for 5 min. The procedure was repeated once. Then, 2 ml of the supernatant was filtered using a 0.22-μm microporous membrane. Phenolic acid contents were analyzed using UPLC (Waters) with a C18 column (1.7 μm, 2.1 × 100 mm; Waters). The detection wavelength, column temperature, and flow rate were 280 nm, 25°C, and 0.3 ml min^−1^, respectively. The mobile phase consisted of water with 0.1% (v/v) formic acid and acetonitrile (A) and 0.1% (v/v) formic acid (B). Gradient elution was performed as follows: 0–6 min, 5–20% A; 6–14 min, 20–95% A; 14–18 min, 95% A.

### Yeast one-hybrid assay

The coding sequences of *SmMYB6*, *SmMYB97*, *SmMYB111*, and *SmMYB112* and the promoters of *SmKSL1*, *SmCPS1*, *SmPAL1*, *SmTAT1*, *SmAOC2*, *SmAOS4*, and *SmJMT2* were amplified using primers listed in Supplementary Data Table S1. The coding sequences of *SmMYB6* and *SmMYB112* were inserted into the XhoI and BamHI restriction sites of the pGADT7-Rec2 vector. The MYB-binding site-containing regions of *SmKSL1*, *SmCPS1*, *SmPAL1*, *SmTAT1*, *SmAOC2*, *SmAOS4*, and *SmJMT2* promoters were inserted into the EcoRI and MluI sites of the pHIS2 vector as described previously [18]. The constructs were co-transformed into yeast strain Y187. Yeast colonies were grown at 28°C for 3 days on SD/−Leu/−Trp medium (DDO), and then screened on SD/−Leu/−Trp/−His medium (TDO) supplemented with 60 mM 3-amino-1,2,4-triazole (3-AT) for 3 days. p53HIS2 and pGADT7-p53 were used as positive controls. p53HIS2 and pGADT7-SmMYBs were used as the corresponding negative controls.

### Transient transformation assay [29]

*SmMYB6*, *SmMYB97*, *SmMYB111*, *SmMYB112, Smi-pri-MIR858a* and the promoters of *SmKSL1*, *SmCPS1*, *SmPAL1*, *SmTAT1*, *SmAOC2*, *SmAOS4* and *SmJMT2* were amplified using the primers in Supplementary Data Table S2. The effector vectors were constructed through insertion of *SmMYB6*, *SmMYB97*, *SmMYB111*, and *SmMYB112* into the pEarleyGate203 vector using the Gateway cloning system (Invitrogen). *Smi-pri-MIR858a* sequence was cloned between the BamHI and SacI sites of the pGPTV-hpt vector. The pEarleyGate203-GFP constructor was used as negative control. Promoters of *SmKSL1*, *SmCPS1*, *SmTAT1*, *SmPAL1*, *SmAOC2*, *SmAOS4*, and *SmJMT2* were cloned into the HindIII and PstI sites of the pGreenII 0800-LUC vector [62] to generate the final reporter vectors. The corresponding effector and reporter vectors were co-transformed into the leaves of tobacco through *Agrobacterium*-mediated transient transformation as described previously [64]. After culture for 3 days, leaves were collected and measured for LUC and *Renilla* signals according to the manufacturer’s recommendations.

## Supplementary Material

Web_Material_uhae047

## Data Availability

All data generated or analyzed during this study are included in this manuscript and its supplementary information files.
