# Supplementary material for: The Smi-miR858a-SmMYB module regulates tanshinone and phenolic acid biosynthesis in Salvia miltiorrhiza
Source: Hortic Res. 2024 Feb 23;11(4):uhae047. doi: 10.1093/hr/uhae047 (PMC11069429; doi:10.1093/hr/uhae047)
Supplement: Web_Material_uhae047 [file web_material_uhae047.pdf]

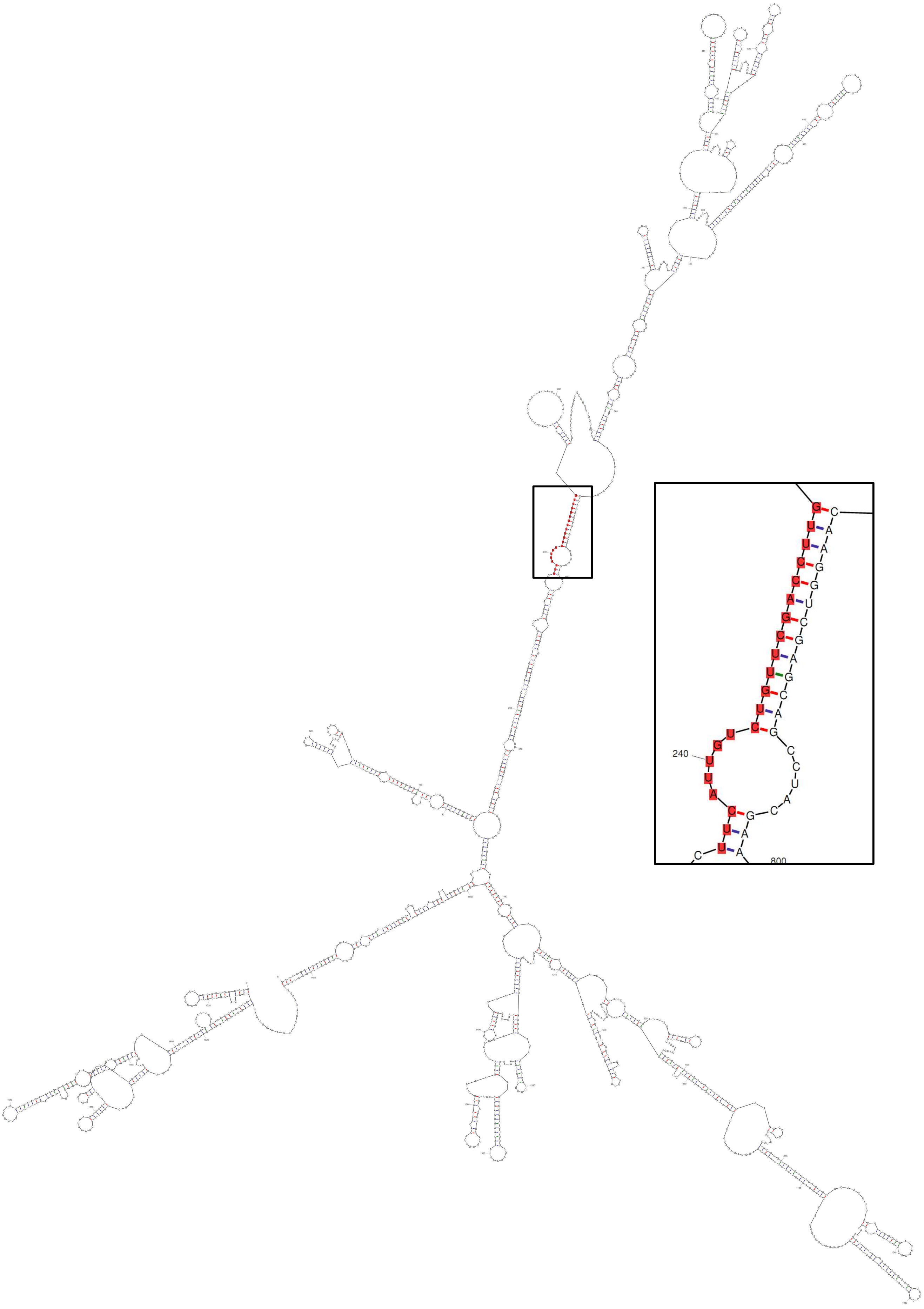

Supplemental Figure S1. The secondary structure of *Smi-MIR858a,* the Smi-miR858a were marked in red color, figure in black box showed the magnifications of indicated region in the secondary structure.

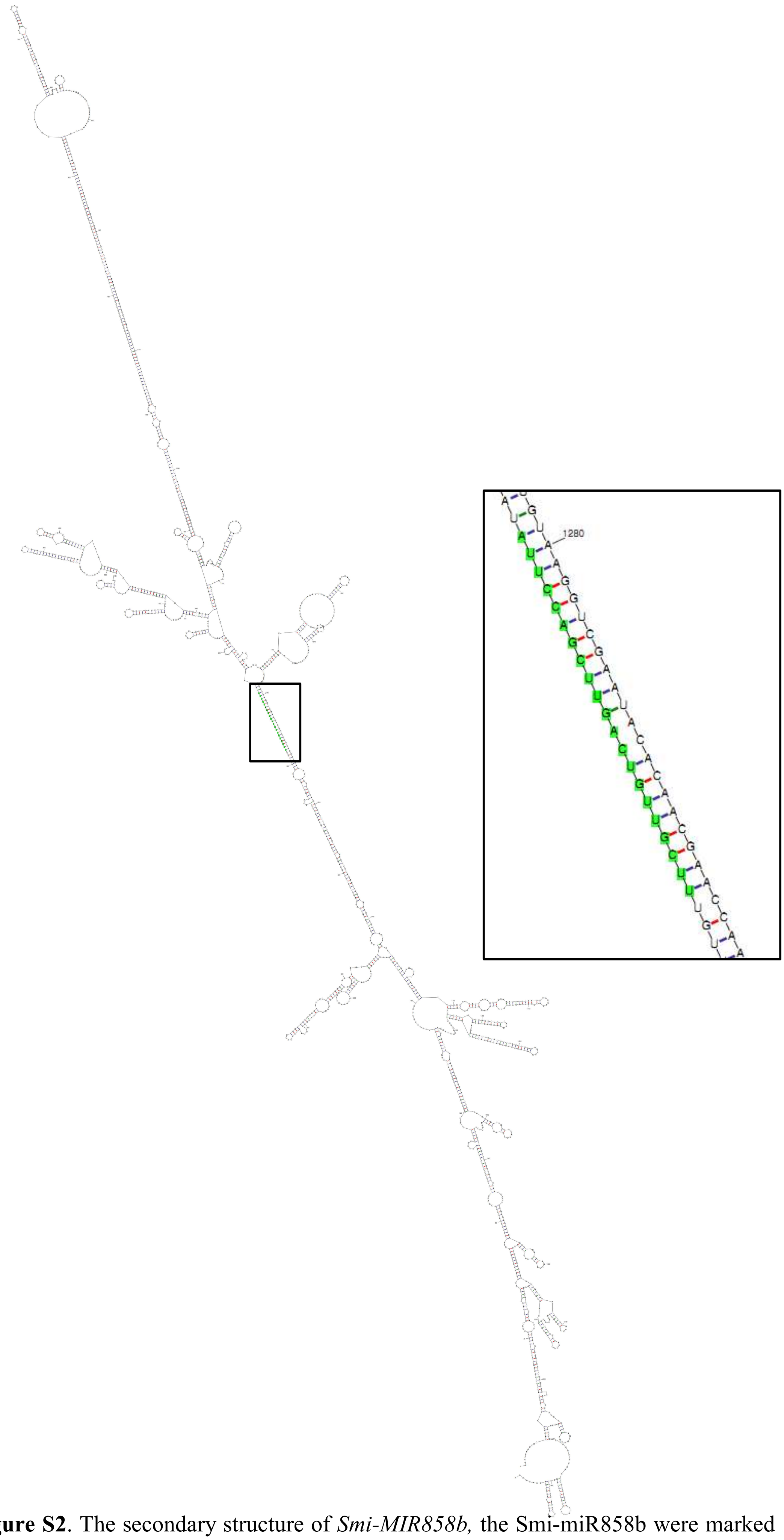

Supplemental Figure S2. The secondary structure of *Smi-MIR858b,* the Smi-miR858b were marked in green color, figure in black box showed the magnifications of indicated region in the secondary

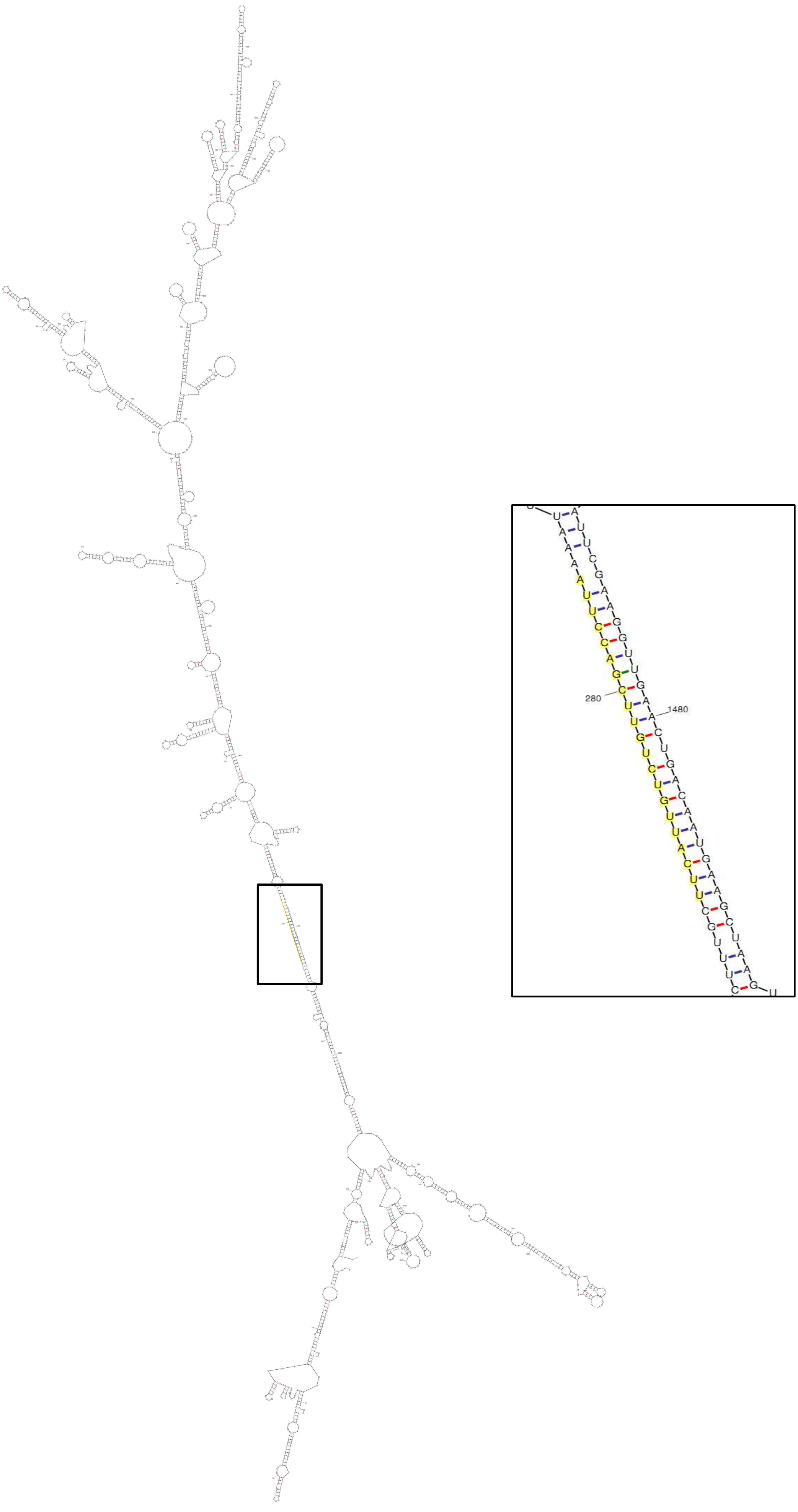

Supplemental Figure S3. The secondary structure of *Smi-MIR858c,* the Smi-miR858c were marked in yellow color, figure in black box showed the magnifications of indicated region in the secondary structure.

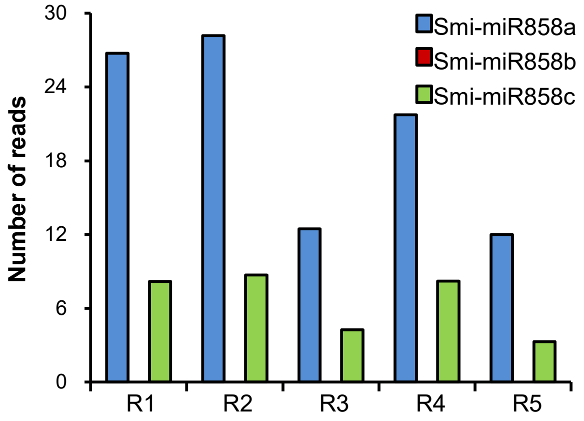

Supplemental Figure S4. Reads of Smi-miR858a. Smi-miR858b and Smi-miR858c from small RNA libraries constructed for roots of *S. miltiorrhiza* plants cultivated *in vitro* for 5 (R1), 10 (R2), 15 (R3), 20 (R4) and 25 (R5) days.

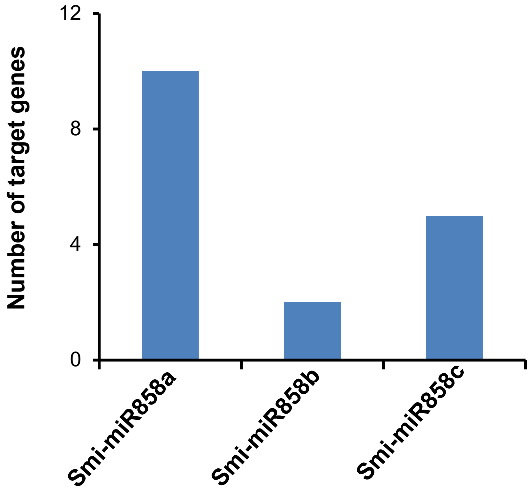

Supplemental Figure S5. The number of target genes predicted for Smi-miR858a, Smi-mi858b and Smi-mi858c.

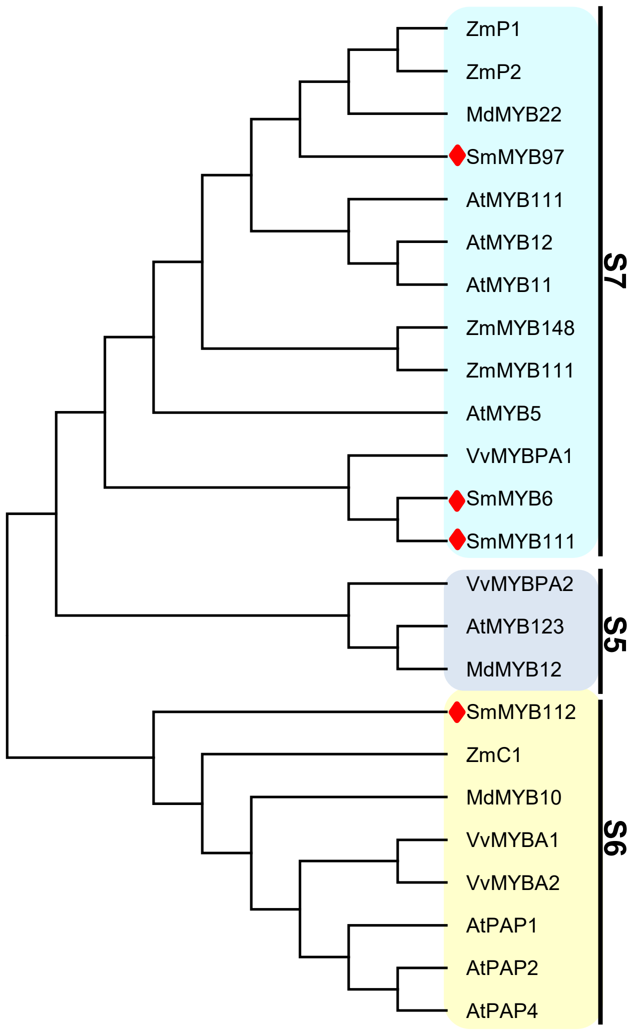

Supplemental Figure. S6 Phylogenetic analysis of SmMYB6, SmMYB97, SmMYB111 and SmMYB112. Phylogenetic analysis of SmMYB6, SmMYB97, SmMYB111, SmMYB112 and their homologs from *A. thaliana*, *Zea mays*, *Malus domestica* and *Vitis vinifera*. SmMYBs are marked with a red diamond. The sequences are listed in Table S4 and were aligned using ClustalW. The neighbor-joining phylogenetic tree was constructed using MEGA6.

```
SmMYB6     1   MGRSACCSKVGLRRGPWSTKEDSLLASYIQQHGEGQWRSLPKKAGLLRCGKSCRLRWMNY 60
SmMYB97    1   MGRAPCCEKVGLRRGRWTAEEDEKLTKYIEENGEGSWRSLPKNAGLLRCGKSCRLRWINY 60
SmMYB111   1   MGRSPCCSKVGLRRGPWSTKEDSLLANYIQQNGEGQWRSLPKKAGLLRCGKSCRLRWMNY 60
AtMYB111   1   MGRAPCCEKIGLKRGRWTAEEDEILTKYIQTNGEGSWRSLPKKAGLLRCGKSCRLRWINY 60
                                          R2 domain

SmMYB6     61  LRPGIKRGNISEDEEDLIVRLHRLLGNRWSLIAGRLPGRTDNEIKNYWNTHLLKKLNTAA 120
SmMYB97    61  LRSDVKRGNISAEEEEIIINLHASKGNRWSLIAAHLPGRTDNEIKNYWNSHLSRKIHSFR 120
SmMYB111   61  LRPGIKRGNISEDEEDLIVRLHGLLGNRWSLIAGRLPGRTDNEIKNYWNTHLLKKLKTAA 120
AtMYB111   61  LRRDLKRGNITSDEEEIIVKLHSLLGNRWSLIATHLPGRTDNEIKNYWNSHLSRKIYAFT 120
                                       R3 domain

SmMYB6     121 A-------------AADKKKIKKP--------PKKSAA----------AAAASQDMKNSK 149
SmMYB97    121 RP---------NPNFIPPPPSSKKATSVNRRGSRKRPG---------HQEDAAVVMPTTP 162
SmMYB111   121 APHKD------LPNLAAKPKKKKPKQKPTPPSPLKDES----------AADEPTPPPKTK 164
AtMYB111   121 AVSGDGHNLLVNDVVLKKSCSSSSGAKNNNKTKKKKKGRTSRSSMKKHKQMVTASQCFSQ 180

SmMYB6     150 VYAPKPMRVS---SRSDSDDSLGS-SDG--APEVLVSA---WP---EVEDP--------- 188
SmMYB97    163 TPERE----AAGRIATEERESGSSISMLGDIVEDLSG---LWGPEFEISRVGPGSE---- 211
SmMYB111   165 VYLPKPIRVSSAFSRSNSYDSLASNSDGEKAAEELSYVPLQWPPIFELEEGDYGVC---- 220
AtMYB111   181 PKELESDFSEGGQNGNFEGESLGPYEWLDGELERLLSS-CVWECTSEEAVIGVNDEKVCE 239

SmMYB6     188 -----------------AALYHFSDSFAD------------------DMWEK-------- 205
SmMYB97    212 NGDTG-----------ETSFKCNEFGLGETGSGLGVSSEIYENTFSWMLDDDCDEIWDS- 259
SmMYB111   220 -----------------AAVGGGSDDFLDGGFILPVINHSDSISSDVNMLEK-------- 255
AtMYB111   240 SGDNSSCCVNLFEEEQGSETKIGHVGITEVDHDMTVEREREGSFLSSNSNENNDKDWWVG 299

SmMYB6     205 ----------VYAEYMQLL------------------------ 214
SmMYB97    259 ------THQGLDDVMFSWILS---------------------- 274
SmMYB111   255 ----------VYDEYLQLI------------------------ 264
AtMYB111   300 LCNSSEVGFGVDEELLDWEFQGNVTCQSDDLWDLSDIGEITLE 342
```

Supplemental Figure S7. Amino acid sequence alignment of SmMYB6, SmMYB97, SmMYB111 and *A. thaliana* AtMYB111 that belong to Subgroup 7. The conserved R2 and R3 domains are underlined in brown and gray, respectively.

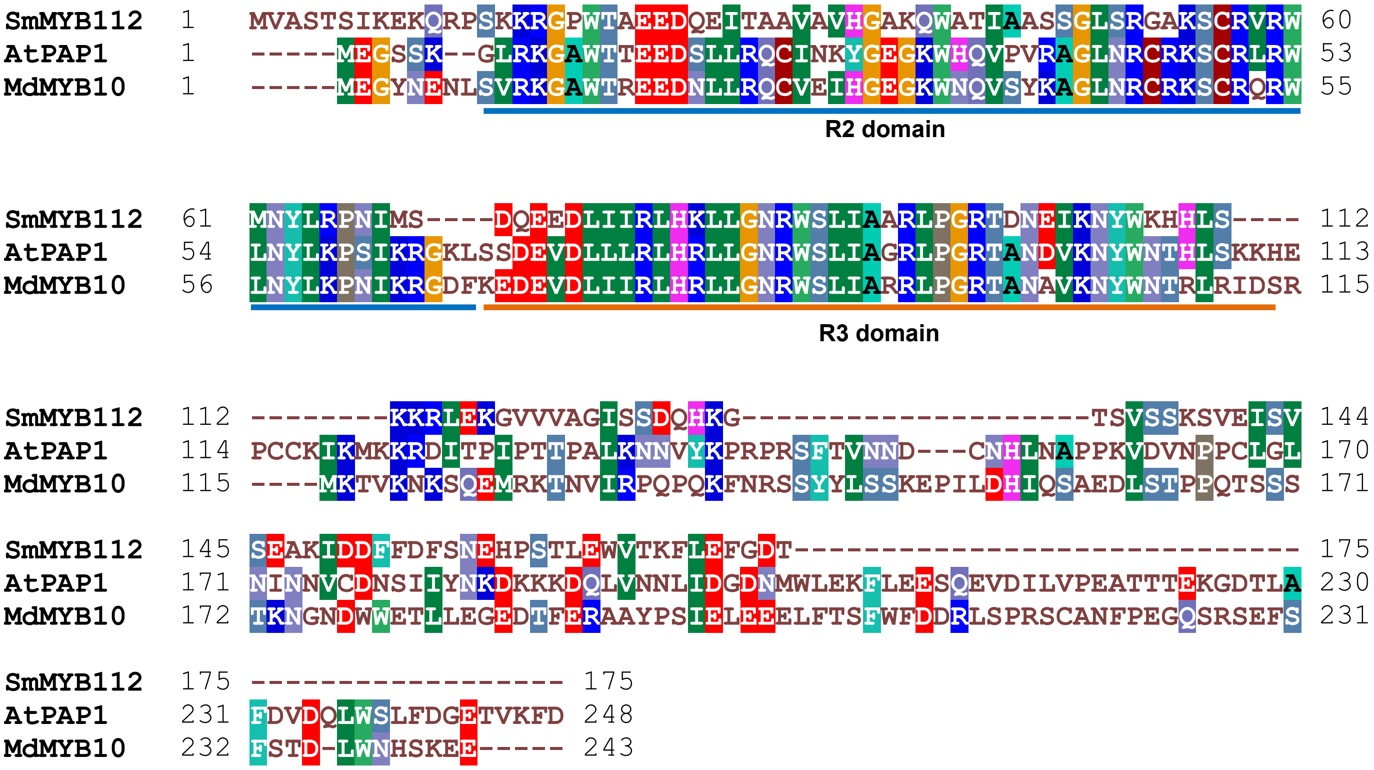

Supplemental Figure S8. Amino acid sequence alignment of SmMYB112 and *A. thaliana* AtPAP1 and *Malus domestica* MdMYB10 that belong to Subgroup 6. The conserved R2 and R3 domains are underlined in blue and orange, respectively.

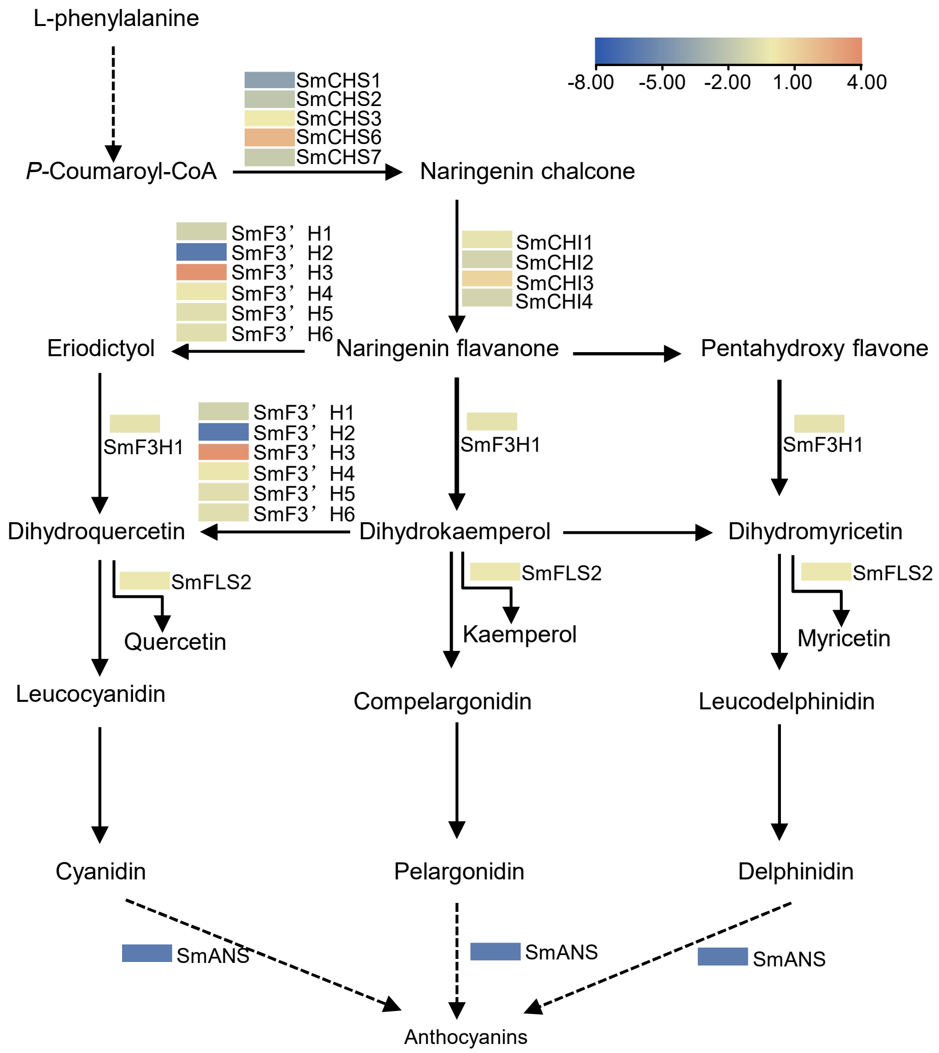

Supplemental Figure S9. RNA-seq analysis of genes involved in flavonoid biosynthesis pathway in transgenic and wild type lines. The average FPKM of three biological replicates was used for comparison.

Table S1 Predicted targets for Sm-miR858a/b/c

miRNA	Target ID	Target name	score
Sm-miR858a	KF059360	SmMYB6	2.0
	KX792200	SmMYB111	2.0
	SMil_00003526		2.0
	KF059451	SmMYB97	2.5
	KF059442	SmMYB88	3.0
	KF059366	SmMYB12	3.0
	KF059445	SmMYB91	3.0
	KF059384	SmMYB30	3.0
	KF059373	SmMYB19	3.0
	SMil_00003712		3.0
Sm-miR858b	KX792200	SmMYB111	2.0
	SMil_00000866		3.0
Sm-miR858c	KF059451	SmMYB97	2.5
	KF059360	SmMYB6	2.5
	SMil_00003526		2.5
	KX792200	SmMYB111	2.5
	KF059442	SmMYB88	3.0

Table S2. Primers used in this study

Primer Name	Sequence (5'-3')
**For artificial microRNA (amiRNA) and overexpressing vector construction**	
1P	CCTTCATTGTCTGTTCGACCTTGTTGTGGCTCTTCCTTTTC
2P	AACAAGGTCGAACAGACAATGAAGGGTAGAGCCAAAACAA
3P	AACATGGTCGAACAGTCAATGATGGATGGAGCTACTAACAG
4P	CCATCATTGACTGTTCGACCATGTTCATCTGTCTCTGCTCC
35SP	CCCACTATCCTTCGCAAGACCCTTCCT
NosP	CTTTATTGCCAAATGTTTGAACGATC
**For stem-loop RT-qPCR**	
qSmi-miR858-F	TCGCTttcattgtctgttcg
qSmi-miR858-R	GTCGTATCCAGTGCAGGGTCCGAGGTATTCGCACTGGATACGACCAA
Universal primer	CAGTGCAGGGTCCGAGGTAT
Sm-5.8s F	GCTCTCGCATCGATGAAGAACGTA
Sm-5.8s R	TTCAAAGACTCGATGGTTCACGGG
**For RT-qPCR**	
qSmMYB6-F	TTAAGAAGCCGCCGAAGA
qSmMYB6-R	GAAGGAATCCGAGAAGTGGTA
qSmMYB97-F	GAAGGCCACGAGCGTTAA
qSmMYB97-R	AAACATGACATCGTCCAAGC
qSmMYB111-F	CGGTAGATTGCCAGGTCG
qSmMYB111-R	GGGGAGGTCTTTGTGAGG
qSmMYB112-F	CATCAGGCTGCATAAACTCCT
qSmMYB112-R	CCACTACCCCTTTCTCCAATC
qSmUbiquitin-F	ACCCTCACGGGGAAGACCATC
qSmUbiquitin-R	ACCACGGAGACGGAGGACAAG
**For yeast one-hybrid assay**	
SmMYB6-*Bam*H1-F	GCATCGATACGGGATCCATGGGAAGATCCGCTTGCTGC
SmMYB6-*Xho*1-R	TTCATCTGCAGCTCGAGAAGAAGCTGCATATACTCGGC
SmMYB112-*Bam*H1-	GCATCGATACGGGATCCATGGTAGCCTCAACTTCGATC
SmMYB112-*Xho*1-R	TTCATCTGCAGCTCGAGAGTGTCACCAAACTCAAGAAA
SmPAL1-*EcoR* I-F	ACTCACTATAGGGCGAATTCCTCGTCCTTGTTTTGCCACT
SmPAL1-*MluI*-R	GGATCGATTCGCGAACGCGTCACGTTTGCGTTGGTTTAGA
SmTAT1-*EcoR* I-F	ACTCACTATAGGGCGAATTCTACGGAGCCGCCCCTAAAA
SmTAT1-*Mlu*I-R	GGATCGATTCGCGAACGCGTGCCGCAACTGAATCGAGAG
SmCPS1-*EcoR* I-F	ACTCACTATAGGGCGAATTCGGGTTGCCAACCAAACTACCG
SmCPS1-*Mlu*I-R	GGATCGATTCGCGAACGCGTCGGAACGGGTTTAGGATTTGA
SmKSL1-*EcoR* I-F	ACTCACTATAGGGCGAATTCGCTCCATGTGTGATGAGTCTTTC

SmKSL1-*Mlu*I-R	GGATCGATTCGCGAACGCGTCATCTTTAGCTCTGGGCGG
SmAOC2-*EcoR* I-F	ACTCACTATAGGGCGAATTCTCCAAAATTTTCAGATCCAC
SmAOC2-*Mlu*I-R	GGATCGATTCGCGAACGCGTGGCTAATTAATTGATTCACCTTTT
SMAOS4-*ECOR* I-F	ACTCACTATAGGGCGAATTCAGATCTCGTCTCAAAAACCA
SmAOS4-*Mlu*I-R	GGATCGATTCGCGAACGCGTTGTCGGTAGATTAGGGATTTGTT
SmJMT2-*EcoR* I-F	ACTCACTATAGGGCGAATTCGGGCTATTACCCCCACCTTA
SmJMT2-*Mlu*I-R	GGATCGATTCGCGAACGCGTTCTCTCTCTCTCTCTCTCTATTCGT
**For transient transformation assay**	
SmMYB6-cacc-F	CACCATGGGAAGATCCGCTTGCTGC
SmMYB6-R	AAGAAGCTGCATATACTCGGC
SmMYB112-cacc-F	CACCATGGTAGCCTCAACTTCGATC
SmMYB112-R	AGTGTCACCAAACTCAAGAAA
SmMYB97- cacc-F	CACCATGGGAAGAGCGCCGTGCTGTGAGAA
SmMYB97-R	TGATAGCAGCCATGAAAACATGAC
SmMYB111- cacc-F C	CACCATGGGAAGATCTCCTTGCTGCTCCA
SmMYB111-R	CAGAAGCTGGAGATACTCATCGTAG
SmPAL1-*Hin*dIII-F	GACGGTATCGATAAGCTTCTCGTCCTTGTTTTGCCACT
SmPAL1-*Pst*I-F	GGATCCCCCGGGCTGCAGCACGTTTGCGTTGGTTTAGA
SmTAT1- *Hin*dIII-F	GACGGTATCGATAAGCTT TACGGAGCCGCCCCTAAAA
SmTAT1- *Pst*I-R	GGATCCCCCGGGCTGCAGGCCGCAACTGAATCGAGAG
SmCPS1- *Hin*dIII-F	GACGGTATCGATAAGCTTGGGTTGCCAACCAAACTACCG
SmCPS1- *Pst*I-R	GGATCCCCCGGGCTGCAGCGGAACGGGTTTAGGATTTGA
SmKSL1- *Hin*dIII-F	GACGGTATCGATAAGCTTGCTCCATGTGTGATGAGTCTTTC
SmKSL1- *Pst*I-R	GGATCCCCCGGGCTGCAGCATCTTTAGCTCTGGGCGG
SmAOC2- *Hin*dIII-F	GACGGTATCGATAAGCTTTCCAAAATTTTCAGATCCAC
SmAOC2- *Pst*I-R	GGATCCCCCGGGCTGCAGGGCTAATTAATTGATTCACCTTTT
SmAOS4- *Hin*dIII-F	GACGGTATCGATAAGCTTAGATCTCGTCTCAAAAACCA
SmAOS4- *Pst*I-R	GGATCCCCCGGGCTGCAGTGTCGGTAGATTAGGGATTTGTT
SmJMT2- *Hin*dIII-F	GACGGTATCGATAAGCTTCAAGCAATCAAGCTCTCTTT
SmJMT2- *Pst*I-R	GGATCCCCCGGGCTGCAGTCTCTCTCTCTCTCTCTCTATTCGT

Table S3 Different expression genes (DEGs) between wild type and overexpression lines

gene_id	WT(FPKM)	OE(FPKM)	P.Value	log_2(fold_change)
SMil_00021054	11.20865	2.799955556	0.004390358	-2.001136694
SMil_00014726	76.96103333	19.22327222	0.007090859	-2.001274235
SMil_00009512	3.771643333	0.939901344	0.011196961	-2.004612014
SMil_00015847	7.42375	1.848782667	0.001906346	-2.005572489
SMil_00010714	16.82589333	4.188296667	0.003830126	-2.006247563
SMil_00000858	949.905	236.212	0.030332431	-2.007700979
SMil_00019836	0.345973333	0.085887667	0.024242722	-2.010137961
SMil_00012132	9.703923333	2.397793333	0.002762729	-2.016860835
SMil_00026896	24.50693333	6.037417778	0.010529078	-2.021186423
SMil_00027502	185.8805667	45.76463333	0.0055709	-2.022070921
SMil_00019047	17.29326667	4.255655556	0.009532734	-2.022757125
SMil_00022518	1460.61	359.3621111	0.001723424	-2.0230608
SMil_00019791	44.77103333	11.00848222	0.007740963	-2.023950043
SMil_00022805	1793.21	440.0163333	0.001699407	-2.026915467
SMil_00021467	357.5716667	87.7038	0.001781605	-2.027521167
SMil_00014070	269.876	66.14502222	0.007476382	-2.028592189
SMil_00009448	16.69671	4.08627	0.004070154	-2.030707417
SMil_00021034	2.551218667	0.624019	0.033398858	-2.031524697
SMil_00003862	4.675996667	1.141008222	0.023191953	-2.034964714
SMil_00013158	664.85	162.0247	0.006853572	-2.036815121
SMil_00015179	1219.649333	297.0911333	0.002410059	-2.037488958
SMil_00022185	132.8387333	32.35671111	0.010308079	-2.037538993
SMil_00022208	3.201156667	0.778816778	0.005439158	-2.039237416
SMil_00022307	0.994316667	0.241078444	0.048687987	-2.044202729
SMil_00016648	1280.998667	310.5650333	0.005511072	-2.044301664
SMil_00018846	59.61406667	14.43959	0.002591425	-2.045623014
SMil_00026781	1429.324	345.9133222	0.002647769	-2.046850502
SMil_00029957	74.28396667	17.96614667	0.008720427	-2.047769839
SMil_00015005	4.527393333	1.093810333	0.009809516	-2.049318055
SMil_00018391	3.491273333	0.843479889	0.00607156	-2.049327735
SMil_00001672	7.040373333	1.697277222	0.021332748	-2.052429709
SMil_00021382	0.579116667	0.139222333	0.048979214	-2.056463358
SMil_00015030	5.922873333	1.416195778	0.02066161	-2.064276513
SMil_00001874	37.2459	8.888072222	0.003288575	-2.067139179
SMil_00016915	105.7684333	25.23189444	0.003760149	-2.067588683
SMil_00018907	49.8009	11.87600778	0.002287486	-2.068121872
SMil_00001432	378.899	90.33892222	0.001326648	-2.068393725
SMil_00013217	74.39556667	17.72824889	0.008758425	-2.069166611
SMil_00003375	16.56466667	3.947022778	0.017756201	-2.069272422
SMil_00014783	3.928273333	0.935592778	0.018797409	-2.069942687
SMil_00005761	5.223016667	1.243290222	0.00460317	-2.070720202
SMil_00026601	1.617616667	0.385049889	0.003281554	-2.070752481
SMil_00003530	341.3916667	81.00232333	0.00471478	-2.075392649
SMil_00007943	57.67356667	13.61172	0.002515828	-2.083060865
SMil_00009775	1.506151333	0.355296778	0.012495126	-2.083770225
SMil_00015445	4.82747	1.137696778	0.019050051	-2.085151196
SMil_00021174	7.976986667	1.878344444	0.0348863	-2.086382223
SMil_00000234	46.28443333	10.89036	0.001900616	-2.087475413
SMil_00012013	6.291986667	1.479166333	0.03230053	-2.08873132
SMil_00002680	10.31925667	2.420569333	0.000641086	-2.091920728
SMil_00021963	52.88346667	12.39197889	0.000795516	-2.093410161
SMil_00021834	5.49184	1.286727444	0.022069895	-2.093583101
SMil_00006054	243.5463667	56.96924444	0.040040527	-2.095941283
SMil_00001190	5040.066667	1178.509222	0.007656525	-2.096479769

SMil_00027012	20.36228	4.754692222	0.005076292	-2.09847525
SMil_00025676	5.740156667	1.339130667	0.018195466	-2.099793373
SMil_00021101	106.0518333	24.70395556	0.002791527	-2.101955594
SMil_00008567	297.1974333	69.13284333	0.019021254	-2.103978489
SMil_00011541	26.97213333	6.27202	0.001283082	-2.10446757
SMil_00001770	5.05594	1.173571778	0.00781454	-2.10707326
SMil_00018806	1.722513333	0.399511556	0.01010514	-2.108206013
SMil_00006569	1.3815	0.320039444	0.041899812	-2.109913931
SMil_00013898	3.375225667	0.781418889	0.019669294	-2.110815929
SMil_00022310	8.732373333	2.013204	0.001029173	-2.116880441
SMil_00013395	10.12454333	2.332596667	0.008697243	-2.117848062
SMil_00010665	4.306873333	0.990524778	0.03412146	-2.120375925
SMil_00017851	137.0152333	31.50565556	0.00017731	-2.120653567
SMil_00013767	0.682336667	0.156767889	0.044060113	-2.121853665
SMil_00027027	18.10166333	4.150523222	0.006827223	-2.124757149
SMil_00005365	4.92369	1.126108778	0.031460078	-2.128393738
SMil_00018535	137.1920667	31.34197333	0.012135902	-2.130029136
SMil_00015983	17.68206667	4.033138889	0.001759726	-2.132311912
SMil_00024799	1736.059667	395.6637444	0.007006178	-2.133469754
SMil_00022585	487.8646667	110.8898889	0.007896101	-2.137353176
SMil_00018619	128.8539	29.26455889	0.005572937	-2.138509766
SMil_00004005	154.3453333	34.92615556	0.000213713	-2.143782109
SMil_00026296	13.88165667	3.137268667	0.021804083	-2.145598765
SMil_00019297	1.727297	0.389236778	0.044947166	-2.149796231
SMil_00005650	18.71303333	4.209754444	0.011189399	-2.152235446
SMil_00000129	9.299726667	2.08857	0.018006178	-2.154672816
SMil_00007660	269.3269667	60.19605556	0.013397436	-2.161617828
SMil_00029167	129.4744667	28.93088922	0.011429062	-2.161985044
SMil_00026950	13.39378667	2.98226	0.005896392	-2.167085949
SMil_00011043	1152.355	256.303	0.010010837	-2.168662958
SMil_00028801	105.4147667	23.43535556	0.000154932	-2.169318388
SMil_00002952	5.356583333	1.188791222	0.017138571	-2.171817711
SMil_00017717	2.821414	0.623296556	0.021905717	-2.17842773
SMil_00019082	18.88927667	4.161286667	0.003278683	-2.182465875
SMil_00013059	263.8886667	58.10577111	0.004069308	-2.183176027
SMil_00006059	25.34846	5.563993333	0.039907689	-2.187705505
SMil_00007054	125.7587333	27.58845556	0.001057456	-2.188521992
SMil_00027134	1880.93	412.0003222	0.001929469	-2.190728788
SMil_00027605	4.98311	1.090187444	0.028022331	-2.192470211
SMil_00025593	72.49626667	15.85915667	0.00082045	-2.192590647
SMil_00022929	0.702801667	0.153448111	7.91435E-05	-2.195366726
SMil_00001282	2.889024	0.630198556	0.00694065	-2.196703836
SMil_00001111	2.188010333	0.476821222	0.049939012	-2.198099198
SMil_00015891	203.7613333	44.20424889	0.005205692	-2.204623353
SMil_00006691	8.77866	1.901696667	0.012762155	-2.206713594
SMil_00004531	5.832283333	1.261632222	0.017632494	-2.208769395
SMil_00015215	76.93993333	16.60221	0.001987968	-2.212357281
SMil_00012558	109.0622333	23.53331333	0.003596537	-2.212375242
SMil_00007357	6.56911	1.417322444	0.048753243	-2.212529911
SMil_00017512	251.4963333	54.22725556	0.015858004	-2.213447304
SMil_00024631	117.9727333	25.33272111	0.007946954	-2.219379495
SMil_00019219	98.44223333	21.07689556	0.017880501	-2.223615002
SMil_00009397	20.15735667	4.315304444	0.015665047	-2.223772213
SMil_00005820	2.310820333	0.494015111	0.039968229	-2.225778017
SMil_00011347	469.2573333	100.2927556	0.003213711	-2.226161891
SMil_00024693	0.96524	0.2059088	0.017730713	-2.228882214
SMil_00016049	263.9343333	56.27891111	0.003496502	-2.229512712

SMil_00015480	7.54772	1.607571667	0.006492825	-2.231157849
SMil_00016739	4.75293	1.012164111	0.004768605	-2.231373928
SMil_00021524	10.62354667	2.261383333	0.013537838	-2.231988017
SMil_00005865	46.69483333	9.931473333	0.00025825	-2.233183266
SMil_00025716	67.32243333	14.30227111	0.007822482	-2.234843067
SMil_00012480	9.820276667	2.078529444	0.024238455	-2.240200485
SMil_00009349	3.796436667	0.802928667	0.021536828	-2.241302213
SMil_00028369	1156.810667	244.6395444	0.002293202	-2.24142323
SMil_00008607	4.343113333	0.917828011	0.021938348	-2.242433858
SMil_00006816	9.269083333	1.956293	0.013046864	-2.244304207
SMil_00005833	153.0157	32.24922556	0.008613882	-2.246343266
SMil_00022077	440.4786667	92.82664333	0.024620511	-2.246461292
SMil_00010588	6.020596667	1.268045778	0.014162537	-2.247299642
SMil_00021732	5.76046	1.213237222	0.010543016	-2.247322357
SMil_00018363	11.51214333	2.422185556	0.011937557	-2.248775165
SMil_00023610	15.85993333	3.329626667	0.00045905	-2.251954377
SMil_00017929	6.04016	1.267662333	0.012364615	-2.25241626
SMil_00024374	1782.166667	373.4085556	0.008965801	-2.254805375
SMil_00007882	2.341707667	0.489708778	0.045236211	-2.257565023
SMil_00008391	1.095175333	0.228932267	0.006190552	-2.258169136
SMil_00017617	1.147197667	0.239491867	0.026879776	-2.260065428
SMil_00014800	37.231	7.768074444	0.012819486	-2.260875435
SMil_00023258	9.221173333	1.922114778	0.024779334	-2.262255848
SMil_00020252	17.25748667	3.594767778	0.007115757	-2.263251889
SMil_00009885	3.7427	0.779088778	0.012127893	-2.264219773
SMil_00013718	4.688533333	0.975100222	0.010457042	-2.265514276
SMil_00024598	14.826	3.080586667	0.021520863	-2.266852388
SMil_00006464	58.41366667	12.13719556	0.00052993	-2.266870838
SMil_00023267	807.5946667	167.3270811	0.010802875	-2.270960425
SMil_00016906	6.71957	1.391531778	0.030553413	-2.27169506
SMil_00010151	1.269078333	0.261598067	0.02086271	-2.278357338
SMil_00011931	12.55357333	2.585031778	0.010616983	-2.27984416
SMil_00028936	1.843136667	0.379201356	0.010594532	-2.281127023
SMil_00004951	2.75537	0.566324111	0.024615607	-2.282546203
SMil_00021627	2.456323333	0.504619222	0.013442298	-2.283233411
SMil_00013400	3.007751667	0.617402667	0.018834737	-2.284401837
SMil_00023430	0.842353333	0.172866556	0.008718286	-2.284766735
SMil_00013979	93.0478	19.07418889	6.31134E-05	-2.286350331
SMil_00022396	635.545	130.2565111	0.000491236	-2.286638789
SMil_00007592	147.8148	30.28625	0.002597671	-2.287055865
SMil_00025375	6.988013333	1.429491111	0.036609896	-2.289380712
SMil_00001218	43.72466667	8.938266667	0.001720984	-2.290380394
SMil_00020281	14.47561	2.958650889	0.016868838	-2.290612766
SMil_00004220	216.4904	44.07921111	0.009968911	-2.296132743
SMil_00021657	261.496	53.12786444	0.007576692	-2.299248251
SMil_00018966	6.05393	1.224461222	0.014559539	-2.305724908
SMil_00008374	131.4716667	26.58043889	0.005463647	-2.306315088
SMil_00000975	18.6865	3.776097667	0.000859372	-2.307028391
SMil_00015959	54.06453333	10.92309444	0.007637458	-2.307300867
SMil_00004558	10.76525	2.174591222	0.016800883	-2.307565688
SMil_00022875	4.883956667	0.986533911	0.010004182	-2.307609852
SMil_00002699	187.21	37.6879	0.000448591	-2.312484186
SMil_00017066	11.75278667	2.365760556	0.031056281	-2.312626903
SMil_00023375	3.274586667	0.657571444	0.001284602	-2.316093259
SMil_00014429	3.386435333	0.679612889	0.00984787	-2.316982328
SMil_00003147	1005.343667	201.4049678	0.042565879	-2.319517583
SMil_00027653	84.24463333	16.87682333	0.021352488	-2.319541409

SMil_00027227	6.685693333	1.33428	0.029275923	-2.325015732
SMil_00030031	22.93392	4.572965556	0.006934691	-2.326281012
SMil_00015019	48.0188	9.56195	0.000602068	-2.328222584
SMil_00001665	27.22075667	5.406696667	0.045462831	-2.331887847
SMil_00017163	43.37386667	8.605473444	0.012066784	-2.33349959
SMil_00011930	3.087688	0.611538	0.014463974	-2.336012927
SMil_00011861	31.64563	6.264525556	0.017977601	-2.336729133
SMil_00011664	2.448478333	0.484169444	0.034216953	-2.33830149
SMil_00015403	5.426681667	1.071180111	0.041817342	-2.340869202
SMil_00026795	7.613053333	1.502218556	0.003071061	-2.341380461
SMil_00012167	1.279317667	0.252420411	0.025898128	-2.341474065
SMil_00020044	2.030608	0.400568444	0.033358263	-2.341791081
SMil_00027759	15.17783667	2.993418889	0.008217366	-2.342100091
SMil_00020838	2.610893333	0.512901222	0.003567207	-2.347790604
SMil_00005328	10.01801	1.962005111	0.014123662	-2.352195252
SMil_00003397	5.472516667	1.070852889	0.026458297	-2.353444143
SMil_00024239	83.13663333	16.21941	0.008218702	-2.357762986
SMil_00002234	5.525023333	1.076991	0.021941768	-2.358974363
SMil_00028036	7.099786667	1.383614222	0.003121182	-2.359333927
SMil_00008067	5.433173333	1.058430778	0.009613441	-2.359868155
SMil_00008373	5.499626667	1.070523622	0.034839719	-2.361017056
SMil_00010420	3.329803333	0.647342333	0.047552057	-2.362836213
SMil_00011117	23.85406667	4.613053333	9.65388E-05	-2.370441362
SMil_00002830	10.86343	2.099519111	0.006122385	-2.371348863
SMil_00010377	193.7233333	37.34787778	1.126E-05	-2.37489956
SMil_00015156	0.769106667	0.148133778	0.028274645	-2.376283054
SMil_00025999	214.854	41.25135556	0.000124525	-2.380843203
SMil_00005927	845.588	161.9257	0.000563251	-2.384622922
SMil_00026806	4.596576667	0.879516889	0.014375206	-2.385776617
SMil_00011286	5.011993333	0.957789889	0.016377671	-2.387603385
SMil_00015525	1.852154333	0.352829111	0.012124925	-2.392162814
SMil_00012362	42.70543333	8.08957	0.001998826	-2.400284709
SMil_00020547	9.32255	1.764545444	0.003800681	-2.401428044
SMil_00018281	1.185590667	0.224088333	0.035793923	-2.40346655
SMil_00000119	7.959273333	1.503554444	0.010539292	-2.404259612
SMil_00011698	6.159297	1.162491667	0.032555699	-2.405545322
SMil_00013497	18.6736	3.520450889	0.003093319	-2.407167963
SMil_00018986	3.565992333	0.670275444	0.025029844	-2.411477614
SMil_00026346	110.8434	20.804253	0.010861065	-2.413572477
SMil_00021954	57.10343333	10.65188111	0.001015113	-2.422469259
SMil_00000953	0.159107	0.029665467	0.043643162	-2.423140926
SMil_00023291	2.904524333	0.540949	0.014218174	-2.424737426
SMil_00029430	1019.071	189.5952222	1.1819E-05	-2.426260055
SMil_00012472	0.370244	0.068727756	0.024595436	-2.429511605
SMil_00023734	2.2277	0.410756889	0.039699554	-2.439198285
SMil_00008606	3.063521667	0.564566889	0.032153371	-2.439974631
SMil_00003532	18.65487667	3.427857778	0.008688552	-2.444173664
SMil_00007497	1.999083667	0.366933556	0.017284647	-2.445748106
SMil_00018309	4.304854	0.787735222	0.023461634	-2.450181619
SMil_00029778	101.3087	18.53046889	0.006374473	-2.45078678
SMil_00011394	288.9183333	52.74913333	3.36657E-05	-2.453442457
SMil_00014428	6.095	1.111275333	0.004471915	-2.455409913
SMil_00012177	3837.96	699.3654444	0.000825328	-2.456221256
SMil_00013434	319.67	58.01886667	0.000734394	-2.461989339
SMil_00002079	3.763086333	0.681695222	0.024164546	-2.464717612
SMil_00021055	1.286733	0.233013456	0.014942557	-2.46522755
SMil_00021084	1.808163333	0.327435222	0.018978957	-2.465243578

SMil_00014562	2.725868	0.493150889	0.007037866	-2.466614662
SMil_00004108	485.3633333	87.6213	0.003410289	-2.4697116
SMil_00001789	5.086093333	0.917266333	0.046937976	-2.471145343
SMil_00028279	17.72868	3.182104667	0.014702629	-2.478031928
SMil_00015010	302.6872667	54.26064333	0.012476249	-2.479849928
SMil_00002292	293.9463333	52.63565567	0.017670599	-2.481440457
SMil_00013689	0.901734	0.161274089	0.01485283	-2.483187253
SMil_00009369	44.58096667	7.970959667	0.011898648	-2.483602566
SMil_00016114	7.52755	1.344066	0.007795709	-2.485576403
SMil_00001976	2.882406667	0.514440111	0.027193042	-2.486198854
SMil_00006570	42.8567	7.63567	0.000201496	-2.488694107
SMil_00010318	8.38252	1.492571444	0.008512122	-2.48958403
SMil_00011496	387.817	68.82945556	0.000983052	-2.494278042
SMil_00005233	2.682046667	0.474027111	0.001643251	-2.500292861
SMil_00010848	8.14983	1.439617	0.003082957	-2.501084922
SMil_00002485	1.867834667	0.329141889	0.039479763	-2.504585208
SMil_00003815	471.927	82.77038889	0.00013243	-2.511377073
SMil_00026146	175.629	30.76395556	2.92182E-05	-2.513218165
SMil_00019103	3.267671	0.571342778	0.01945561	-2.515834278
SMil_00008249	11.03940667	1.927599667	0.00337116	-2.517785272
SMil_00009979	11.61096667	2.023046056	0.036229124	-2.520887019
SMil_00025215	11.82313333	2.057624444	0.004852644	-2.522560833
SMil_00000319	10.23723667	1.774529111	0.000733375	-2.528318195
SMil_00027823	4.500016667	0.778770222	0.035869731	-2.530660719
SMil_00015830	23.20844667	4.007536667	0.002307332	-2.533862341
SMil_00020268	191.1438667	32.94826778	0.002975854	-2.536384384
SMil_00013381	3.642706667	0.627905667	0.033373731	-2.536391088
SMil_00019357	6.591483333	1.135831778	0.005865182	-2.536853982
SMil_00008411	6.421623667	1.105768556	0.038664816	-2.537888668
SMil_00021047	5.069223333	0.870290778	0.003059326	-2.542195312
SMil_00024981	2.529292	0.434124444	0.001521252	-2.542553037
SMil_00013214	3.492657	0.599418333	0.019879676	-2.542689854
SMil_00026652	2.742053333	0.469738178	0.019527447	-2.545327874
SMil_00007576	257.2656667	43.96698889	3.95515E-05	-2.548766298
SMil_00016986	280.3833333	47.89350333	0.004294535	-2.549498719
SMil_00000875	2.607673333	0.444783	0.011104339	-2.551589599
SMil_00020977	31.41653333	5.347717111	0.012314637	-2.554528939
SMil_00009482	418.4030333	70.82817667	0.012090436	-2.562498002
SMil_00024796	589.169	99.22133333	0.004909393	-2.569959273
SMil_00010985	16.27644333	2.739008444	0.001761005	-2.571059863
SMil_00022770	29.02901667	4.853369444	0.018354046	-2.580437114
SMil_00007534	53.00527667	8.856468	0.017676048	-2.581332622
SMil_00002142	12.70878333	2.105821111	0.005243863	-2.593371131
SMil_00024168	1.973583333	0.326466556	0.001126857	-2.595810328
SMil_00022339	67.31923333	11.13376667	0.002144685	-2.596076993
SMil_00015340	2.256708333	0.372103667	0.003464604	-2.600443459
SMil_00021502	6.54168	1.076048667	0.020949481	-2.60391786
SMil_00018265	5.091296667	0.83435	0.005622428	-2.609308524
SMil_00007259	278.556	45.56913333	0.003583228	-2.611838554
SMil_00025910	0.82962	0.134878211	0.008520175	-2.620793367
SMil_00009781	2.982515	0.482244556	0.016738493	-2.628692538
SMil_00006586	2.13102	0.344035111	0.005622763	-2.630916419
SMil_00006153	1.879703667	0.303048667	0.031653159	-2.63288384
SMil_00026401	4.84408	0.779381667	0.001195394	-2.635820791
SMil_00002303	5.059746667	0.806852889	0.004889562	-2.648687593
SMil_00012951	1.537003333	0.244978844	0.009268801	-2.64939122
SMil_00000210	211.039	33.60331956	0.003915724	-2.65083397

SMil_00003154	329.0527	52.36425556	0.004153347	-2.651664407
SMil_00022723	8.347588667	1.327498333	0.020185808	-2.652649463
SMil_00001303	12.48124333	1.982817778	0.008674929	-2.654137653
SMil_00009223	17.50782667	2.777358889	0.001651002	-2.656214487
SMil_00023640	4.047794	0.638444	0.033506806	-2.664503883
SMil_00025263	4.870733333	0.768114556	0.023728931	-2.664745606
SMil_00007090	66.27306667	10.39499	0.003437067	-2.672534309
SMil_00020564	16.51477667	2.588163222	0.001122208	-2.673756951
SMil_00023574	93.7222	14.68207	8.67898E-05	-2.674335434
SMil_00021719	55.04066667	8.574885667	0.003508894	-2.682308604
SMil_00003637	6.216933333	0.965754333	0.002475194	-2.686474957
SMil_00000213	2.60801	0.404662556	0.001919621	-2.688158137
SMil_00011961	6.045753333	0.937114333	0.03697846	-2.689625137
SMil_00008395	58.31666667	9.030647778	0.003580148	-2.691006876
SMil_00011237	6.892406667	1.066107667	0.014721724	-2.692654682
SMil_00001118	11.58501667	1.781963222	0.022561577	-2.700720653
SMil_00028734	10.79199667	1.655629	0.000131927	-2.704510479
SMil_00025190	2087.693333	319.0363333	6.97785E-05	-2.710117167
SMil_00000533	2.751801667	0.419447222	0.024008861	-2.713815296
SMil_00002532	358.7943333	54.53838556	0.003297727	-2.717813206
SMil_00030197	34.7901	5.275831	0.002366892	-2.721206569
SMil_00016429	2323.56	351.8348667	0.001517629	-2.723366534
SMil_00006292	113.4202	17.1333	3.12445E-06	-2.726802648
SMil_00026006	0.688542667	0.103782967	0.012447825	-2.729976375
SMil_00002829	146.295	22.02876333	0.000702117	-2.731420051
SMil_00006155	22.16049667	3.336174333	0.008005519	-2.731723631
SMil_00026745	5.333763333	0.802181556	0.002645118	-2.733153112
SMil_00005524	275.1645667	41.37726667	0.008140778	-2.733384453
SMil_00009441	8.787523333	1.320206222	0.007588504	-2.734693312
SMil_00009898	2.700555333	0.405558778	0.020865359	-2.735273184
SMil_00013414	297.551	44.52962222	4.34516E-05	-2.740299689
SMil_00011566	11.17550333	1.667971778	0.003385789	-2.744173027
SMil_00016450	3.10096	0.462252	0.004088483	-2.74596345
SMil_00008308	3.301679	0.491424	0.010620376	-2.748159641
SMil_00015798	557.6223333	82.73526889	0.006097456	-2.752713977
SMil_00021203	2.31776	0.343267778	0.021987692	-2.755324841
SMil_00018793	5.046106667	0.743624889	0.022593785	-2.76252374
SMil_00007629	26.3348	3.877365556	0.000407039	-2.76382184
SMil_00025035	279.2176667	41.06194	0.005025011	-2.765516531
SMil_00024632	50.65193333	7.41746	7.75731E-05	-2.771620189
SMil_00012688	3.285404333	0.479560889	0.00670668	-2.776285025
SMil_00000768	2.290516667	0.332886	0.001528041	-2.782572958
SMil_00016524	8.141756667	1.173086	0.001249234	-2.795031322
SMil_00021600	0.876707333	0.126075744	0.003930237	-2.797804572
SMil_00004365	132.1853	18.93839556	0.000383518	-2.80317573
SMil_00004049	6.066496667	0.865031111	0.001669286	-2.810039691
SMil_00010769	1.96072	0.279367222	0.000220271	-2.81114886
SMil_00022472	9.406016667	1.335036667	0.000613298	-2.816704523
SMil_00002784	2.02899	0.287928222	0.001968094	-2.816980643
SMil_00022298	1.892473667	0.268499522	0.007413838	-2.817281799
SMil_00024124	15.13562	2.1421	0.010109645	-2.820850037
SMil_00011615	3.262329333	0.461697667	0.012934448	-2.820882084
SMil_00010658	5.736056667	0.811305667	0.013420997	-2.821741806
SMil_00001678	3.103342333	0.438927889	0.00572216	-2.821767006
SMil_00013661	1.272646	0.179957	0.010495891	-2.822107047
SMil_00002524	6.099355	0.855461333	0.024193141	-2.833882136
SMil_00021665	2.67728	0.374615889	0.022178756	-2.837284033

SMil_00016408	12.60882	1.763784444	0.000120315	-2.837687105
SMil_00003759	49.82986667	6.908045556	0.000732495	-2.850661212
SMil_00023473	162.4778667	22.509	0.010487491	-2.851669333
SMil_00026135	2776.443333	383.7017778	3.93903E-05	-2.855180597
SMil_00007840	156.361	21.56453778	0.003852391	-2.858148018
SMil_00015855	10.33033	1.415392111	0.001798647	-2.867612653
SMil_00021206	4.439975333	0.604582222	0.01377718	-2.876541199
SMil_00010078	5.22269	0.708387444	0.004972398	-2.882182526
SMil_00007306	8.167595667	1.102326889	0.010701562	-2.889359337
SMil_00026897	1.554779333	0.209183333	0.007409109	-2.893870021
SMil_00011208	2.49638	0.335813333	0.034044787	-2.89410614
SMil_00011571	11.12259	1.495555444	0.024493987	-2.894739471
SMil_00009447	17.9447	2.403974667	0.004960021	-2.900064206
SMil_00011958	6.08741	0.814487778	0.000160574	-2.901863583
SMil_00023634	36.527	4.864777778	0.029261672	-2.908517459
SMil_00027209	6.73684	0.895724889	0.010581036	-2.910944437
SMil_00019466	13.35576667	1.770936111	0.002912445	-2.914878723
SMil_00022409	7.396816667	0.9801552	0.019825417	-2.915822406
SMil_00007433	3.92741	0.520238889	0.047124072	-2.916332063
SMil_00025870	29.71436667	3.921983333	0.011761312	-2.921505322
SMil_00003181	10.84665333	1.429654556	0.003168228	-2.923511478
SMil_00027716	8.669126667	1.137609667	0.043069042	-2.929881034
SMil_00003222	213.5093333	27.94575111	0.00150199	-2.93359828
SMil_00003390	1.210257	0.157201444	0.022094592	-2.94462706
SMil_00010307	2.016806667	0.261518333	0.000687851	-2.947088799
SMil_00026544	27.63216667	3.552350444	0.006510883	-2.959502872
SMil_00013056	2.664243333	0.341486556	0.00612296	-2.963825169
SMil_00022624	0.753416667	0.095895	0.017365889	-2.973920448
SMil_00013934	14.64441667	1.860285778	0.007629961	-2.976754556
SMil_00011843	4.040766667	0.511988111	0.002742383	-2.980446831
SMil_00019589	0.39686	0.050198544	0.017790509	-2.982912721
SMil_00022570	22.90647	2.887789778	0.001499826	-2.987717522
SMil_00028968	14.01481	1.763033333	0.020631728	-2.990820529
SMil_00004525	0.773092333	0.097052467	0.005445621	-2.993803945
SMil_00013220	14.76436	1.843482889	0.000915899	-3.00161289
SMil_00000978	3.351876333	0.417019778	0.008998413	-3.00678121
SMil_00007833	317.0463333	39.43865556	6.41556E-06	-3.007011415
SMil_00026769	170.5327667	21.1908	0.040257029	-3.008539011
SMil_00006935	0.111194333	0.013793089	0.042358984	-3.011065786
SMil_00001507	544.2666667	66.91623333	0.000490553	-3.023885537
SMil_00019164	1.091482667	0.134147	0.007996515	-3.024402523
SMil_00019651	1.184186667	0.145384	0.01334103	-3.025956105
SMil_00014430	6.786663333	0.831935111	1.32999E-05	-3.028159536
SMil_00018186	9.177193333	1.119212689	0.003106474	-3.035568777
SMil_00011342	12.15549333	1.480947	0.001403003	-3.037016531
SMil_00009208	17.82205333	2.162565889	0.003582858	-3.042847569
SMil_00007069	2.340881	0.283481189	0.007896446	-3.045726686
SMil_00029751	128.2268667	15.52257133	0.001889487	-3.046259107
SMil_00006130	3.252746667	0.393060778	0.001183763	-3.048834151
SMil_00018089	1.901613333	0.229675556	0.013628324	-3.049554701
SMil_00003526	12.79773667	1.540622333	0.015004151	-3.054303537
SMil_00029404	7.87196	0.944051889	0.000713346	-3.059784827
SMil_00023814	4.203600333	0.500920344	0.028879695	-3.068972397
SMil_00026549	2.694116667	0.319045333	0.018018373	-3.07797899
SMil_00019792	31.84753	3.767238889	0.002952525	-3.07960206
SMil_00019049	4.10553	0.483931	0.007886418	-3.084695213
SMil_00018976	144.4221667	16.98177933	0.024978611	-3.088232656

SMil_00020918	162.1241	19.04217889	0.005033607	-3.089828093
SMil_00027739	70.54373333	8.279413333	2.5966E-05	-3.090917479
SMil_00022184	41.66336667	4.886198889	0.011808111	-3.091994929
SMil_00003973	2.997958	0.350786667	0.001365762	-3.095314355
SMil_00018469	684.9873333	79.77422222	0.001242108	-3.102082767
SMil_00013529	0.266935667	0.031055667	0.038264336	-3.103563641
SMil_00022504	4.624037667	0.535214667	0.006495137	-3.110963594
SMil_00010958	1.320213333	0.152735533	0.001327097	-3.11166343
SMil_00002498	42.42773333	4.876717556	0.006005031	-3.121025282
SMil_00020756	1.635418	0.187681978	0.020666145	-3.123297398
SMil_00018497	56.3989	6.462414444	0.000975733	-3.125521844
SMil_00012008	32.54521	3.661914222	0.00637672	-3.151775321
SMil_00008768	1277.477333	143.7292111	0.000604242	-3.151872487
SMil_00009595	2.86356	0.320648444	0.017191289	-3.158745519
SMil_00001700	3.642555667	0.404331333	0.006893761	-3.171341107
SMil_00012572	4.518094333	0.499972889	0.020902452	-3.175792621
SMil_00030001	1.036243333	0.114384556	0.046349906	-3.179398646
SMil_00029312	7.377087	0.809102778	0.030027499	-3.188656369
SMil_00010512	51.85268667	5.661826889	0.014952706	-3.195079198
SMil_00016300	166.5861667	18.13115556	0.001011085	-3.199725824
SMil_00028606	10.61115667	1.145655889	0.003782385	-3.211336241
SMil_00017097	3.222680667	0.344988333	0.008162249	-3.22364176
SMil_00030081	96.05313333	10.25935778	0.000166313	-3.226892254
SMil_00003811	2.294256667	0.243530111	0.003847702	-3.23585473
SMil_00025376	2.753926667	0.291306667	0.01916198	-3.240879519
SMil_00016859	1185.887667	125.0453111	1.77325E-05	-3.245444491
SMil_00004230	5.609836333	0.589042778	0.016853377	-3.251514366
SMil_00000791	43.1898	4.53166	0.000147643	-3.252579108
SMil_00026918	1.165813333	0.121948778	0.005690488	-3.256989603
SMil_00020904	178.156	18.52989111	0.001356664	-3.265214763
SMil_00018074	12.79699333	1.325219778	0.001891966	-3.271501343
SMil_00020406	11.80306667	1.218625556	0.030313273	-3.275834943
SMil_00003198	2.886875667	0.297350444	0.001362656	-3.279272837
SMil_00017623	0.59967	0.061232878	0.002693947	-3.291790408
SMil_00014517	10.06547	1.025083	0.002995168	-3.295601906
SMil_00000593	9.76336	0.987450222	0.002277387	-3.3055978
SMil_00015198	39.54406667	3.993021111	0.003673609	-3.307908639
SMil_00021274	49.39498333	4.98191	0.003743979	-3.309593662
SMil_00000716	3317.82	333.0107778	1.17565E-06	-3.316594843
SMil_00006892	40.29796667	4.042373333	0.013351857	-3.317432573
SMil_00015731	11.32485333	1.120721556	0.005745648	-3.336992578
SMil_00017087	1.696363333	0.166894667	0.02756076	-3.345435447
SMil_00026185	14.24345333	1.385385889	0.002428171	-3.361939182
SMil_00022057	1.424836667	0.137136311	0.007072701	-3.377114023
SMil_00023834	0.934233333	0.088665022	0.004262639	-3.397345933
SMil_00005256	1.494613333	0.141356244	0.010824869	-3.402364777
SMil_00013083	1472.314333	139.2194111	0.000185848	-3.402653431
SMil_00024966	2.705023333	0.253303178	0.000330333	-3.416703957
SMil_00002774	1.871899	0.174989378	0.001113117	-3.41916334
SMil_00016583	149.2133667	13.68417	0.002302892	-3.446796942
SMil_00023358	2.697942667	0.244288	0.034239156	-3.465204788
SMil_00001482	2.011083667	0.175096389	0.000334843	-3.521751867
SMil_00014553	23.14429	2.007474111	0.000198248	-3.527203018
SMil_00023201	0.372829333	0.031790444	0.029853057	-3.551850281
SMil_00019338	2.775139667	0.234364778	0.003178324	-3.56573271
SMil_00006597	680.378	56.14051444	0.0002499	-3.599222303
SMil_00028637	2659.493333	217.7118889	9.56859E-07	-3.610659322

SMil_00009694	0.952143333	0.077590889	0.004038165	-3.617219611
SMil_00009894	1.366502667	0.111339633	0.00010342	-3.617449133
SMil_00015742	13.43100667	1.092192778	0.008148749	-3.620268013
SMil_00021993	5.429718333	0.439981556	0.009597432	-3.625362409
SMil_00018975	92.59413333	7.467489778	0.000745377	-3.632225526
SMil_00009849	0.895753333	0.069812111	0.00339887	-3.681552263
SMil_00016371	1.213809	0.094573467	0.006940456	-3.681962132
SMil_00030359	1.01974	0.078718	0.027614427	-3.695363983
SMil_00024885	1104.097	83.51755556	5.86542E-06	-3.724643628
SMil_00024037	9.15775	0.684346111	0.000784907	-3.742195117
SMil_00011873	6.99544	0.521049556	2.42869E-05	-3.746922308
SMil_00030254	3.07016	0.227770856	0.004598479	-3.752658779
SMil_00003940	10.17559	0.750166111	0.000264472	-3.761758546
SMil_00028636	1425.419	104.2994	6.90622E-07	-3.772583296
SMil_00028608	4.733046667	0.338965556	0.022774174	-3.803558562
SMil_00027598	4.8574	0.338257111	0.000339456	-3.843992126
SMil_00010248	118.3113333	8.137044333	0.001013543	-3.861939618
SMil_00022715	98.52783333	6.733812111	0.001812351	-3.87103596
SMil_00012146	4.211613	0.279762222	0.000784103	-3.912099809
SMil_00025093	4.242796667	0.281512444	0.022570675	-3.913744936
SMil_00028526	4.718283333	0.311916	0.015663567	-3.919032591
SMil_00013998	9.610073333	0.631901778	0.045798651	-3.92677521
SMil_00028120	12.14746	0.785835044	0.02242595	-3.950284365
SMil_00011216	11.869858	0.760810667	0.006206318	-3.963621393
SMil_00005255	0.82866	0.052786444	0.022927717	-3.972540886
SMil_00007165	911.2183333	57.99459444	0.000168589	-3.973806433
SMil_00014285	38.53143333	2.430688889	0.01555286	-3.986598698
SMil_00009209	39.3854	2.452023667	0.001962293	-4.005616119
SMil_00018977	2.04756	0.127466	0.042418102	-4.005721346
SMil_00003727	6.744923333	0.418164889	0.000587653	-4.011658208
SMil_00028586	1.704303333	0.103976111	0.001092835	-4.034858123
SMil_00007268	3.572766667	0.216699667	0.011901139	-4.043272857
SMil_00001851	187.2963333	11.31655178	0.000620514	-4.048816324
SMil_00006923	2.437573333	0.146916889	0.012956585	-4.052373466
SMil_00020723	9.71961	0.576970778	9.39688E-06	-4.07432827
SMil_00017796	2.784533333	0.163523444	0.010320842	-4.089866167
SMil_00013776	2.22849	0.128949778	0.035034675	-4.111185295
SMil_00026609	1915.773333	110.4925222	3.66618E-06	-4.115906236
SMil_00021462	3.7094	0.212340889	0.011526548	-4.126731736
SMil_00008344	0.613985333	0.035124444	0.019601329	-4.127656882
SMil_00009181	1.25051	0.070865222	0.018103003	-4.141294999
SMil_00016012	958.1106667	53.80797778	4.02068E-07	-4.154300311
SMil_00017101	2.591003333	0.143966	0.010427435	-4.169710831
SMil_00024925	664.9023333	36.26969111	0.000243184	-4.196306077
SMil_00011290	0.453656667	0.024161909	0.031004679	-4.230794517
SMil_00020731	1.18718	0.061798	0.012142627	-4.263834734
SMil_00010467	4.65739	0.234258811	0.000450891	-4.313346478
SMil_00019885	1799.21	90.1559	4.47995E-07	-4.318797867
SMil_00016736	69.53396667	3.368013222	0.000306249	-4.367748185
SMil_00026385	2.433356667	0.117559111	0.008520259	-4.371489537
SMil_00012227	2.307892667	0.110120867	0.047022238	-4.389416356
SMil_00000981	0.864604333	0.039502667	0.008480967	-4.452018115
SMil_00011848	10.69716	0.487220689	5.18698E-05	-4.456508619
SMil_00006159	3.69445	0.163256667	0.009515171	-4.500145792
SMil_00013997	16.47582333	0.707354444	0.001993958	-4.541773443
SMil_00012897	1755.746667	74.58981111	4.11597E-07	-4.556962313
SMil_00023665	37.25126667	1.552535444	3.14124E-05	-4.584591372

SMil_00023780	30.74880667	1.235286667	0.002412431	-4.637612637
SMil_00017663	7.331746667	0.285675444	0.009109071	-4.681707999
SMil_00004555	2.96131	0.114323667	0.011535701	-4.695039526
SMil_00014199	1.928603333	0.068745	0.048099279	-4.81015785
SMil_00011259	9.122833333	0.320223333	0.047156606	-4.832331621
SMil_00000762	1.66491	0.057966	0.046665258	-4.844093447
SMil_00005257	5.2191	0.175152222	0.044522422	-4.897119847
SMil_00026124	5.1278	0.164259667	0.004355766	-4.964289813
SMil_00026221	1.494876667	0.046677111	0.040640896	-5.001167377
SMil_00022642	0.718619333	0.021645844	0.002861816	-5.05306576
SMil_00013013	4.66677	0.134422322	0.000239095	-5.117579729
SMil_00021673	8.098666667	0.215361111	0.033330832	-5.232854744
SMil_00011884	91.43666667	2.323333333	0.031547594	-5.29850475
SMil_00019500	21.42456667	0.533265556	0.002754601	-5.328268069
SMil_00018863	3.979866667	0.086246444	0.026135498	-5.528111306
SMil_00018744	0.988356667	0.020758789	0.001398644	-5.573237577
SMil_00027127	6.577883333	0.136746333	0.012353626	-5.588049366
SMil_00005822	10.66673333	0.201931111	0.022384551	-5.72311141
SMil_00003398	2.559036667	0.048420667	0.002246144	-5.723834066
SMil_00029849	17.04233667	0.316026667	1.01676E-06	-5.752933046
SMil_00002872	2.955423333	0.053904333	0.021466117	-5.776819731
SMil_00028360	13.03143333	0.235391111	0.015494609	-5.790792123
SMil_00013188	6.271453333	0.095724667	0.002054373	-6.033765267
SMil_00022498	4.278223333	0.061523889	0.048141003	-6.119721288
SMil_00000404	4.232666667	0.060253222	0.01637585	-6.134384671
SMil_00024762	2.081063	0.027856222	0.024364765	-6.223177213
SMil_00016511	1.145993333	0.013073889	0.01178083	-6.4537665
SMil_00025037	10.68426	0.072470556	0.039147593	-7.20387632
SMil_00020726	2.745663333	7.21109E-05	5.69776E-05	-15.21657809
SMil_00021843	0.065041667	33.44423778	0.000291485	9.006177724
SMil_00004066	0.001302343	0.37145135	0.030534284	8.155919627
SMil_00024166	0.042061667	2.066828333	0.008270301	5.618768739
SMil_00007262	1.629596667	78.54355667	0.035876722	5.590906089
SMil_00026140	0.083935333	4.010433222	0.012246657	5.578336026
SMil_00016969	0.102438333	3.966458778	0.007431075	5.275023965
SMil_00001194	0.25099	9.610436667	0.000908718	5.258900194
SMil_00014851	0.632209977	20.91402	0.000398974	5.047922783
SMil_00019441	0.182796	5.851467778	0.030534869	5.000492148
SMil_00000490	0.057025333	1.771662333	0.013107674	4.957356877
SMil_00014654	0.236995333	6.918465111	0.029953758	4.867521449
SMil_00025299	0.061560867	1.652369556	0.041404616	4.746379032
SMil_00023493	3.4867	89.31447778	0.019243329	4.67895991
SMil_00005751	0.268125333	6.774356667	0.029474443	4.659104505
SMil_00025317	0.074179	1.794381333	0.018905567	4.596331888
SMil_00014698	0.479386667	10.76176556	0.013067157	4.48858119
SMil_00002456	0.067828333	1.427464556	0.023926311	4.39542307
SMil_00015884	0.332494667	6.545263333	0.006947997	4.299048132
SMil_00026928	0.062352333	1.195637111	0.026014655	4.261192223
SMil_00010901	0.032585267	0.571135222	0.041855914	4.131540652
SMil_00005555	0.03637	0.631135333	0.014762632	4.117128562
SMil_00010860	0.137216	2.268042222	4.97611E-05	4.046926877
SMil_00018206	0.181064333	2.813806667	1.55406E-05	3.957948914
SMil_00017197	0.280985	4.289691111	0.013461278	3.932308746
SMil_00029152	1.456128667	22.11100556	0.000344056	3.924554891
SMil_00004329	0.279266667	4.181945667	0.002507069	3.904459029
SMil_00021057	0.057628667	0.855068778	0.046610517	3.891181922
SMil_00015883	0.986469	14.22911222	0.016571916	3.850428128

SMil_00029905	0.133169333	1.918558333	0.039364099	3.848688833
SMil_00015646	0.410219667	5.823877111	0.01615392	3.82751135
SMil_00023840	0.250881333	3.496617	0.038686167	3.800882743
SMil_00005898	0.099328	1.360483889	0.001034615	3.775775599
SMil_00001069	0.888811667	11.88249333	0.000591812	3.740816028
SMil_00004760	0.110203	1.438024556	0.009266461	3.705852908
SMil_00000920	0.443686	5.761738889	6.29178E-05	3.698893345
SMil_00022611	0.782443333	10.11130667	0.010978123	3.691839364
SMil_00027989	0.121212667	1.563339	0.028201993	3.689018279
SMil_00004416	0.563541667	7.217067778	0.002784462	3.678818615
SMil_00014933	0.05104	0.648752889	0.015555276	3.667968824
SMil_00003425	0.240302333	2.990163889	0.014714774	3.637301998
SMil_00000872	0.454716667	5.48715	0.021211389	3.593017226
SMil_00011862	8.482143333	98.93138556	0.002631996	3.543927515
SMil_00005181	5.127636667	58.42620333	0.008807377	3.510249598
SMil_00025074	0.927052667	10.48814444	0.048927194	3.499964348
SMil_00029646	0.402324	4.394155556	0.018529778	3.449156236
SMil_00020438	0.060932333	0.638365333	0.039456904	3.389102415
SMil_00009511	1.909085	19.81580778	0.015797583	3.375698535
SMil_00017159	0.584183667	5.995033333	0.005101931	3.359273848
SMil_00016187	0.762926333	7.622945556	0.00794155	3.320732907
SMil_00001509	0.63943	6.376491111	0.012108385	3.317904411
SMil_00015220	0.722158667	7.12026	0.000256559	3.301542169
SMil_00003558	0.049795333	0.490039222	0.044965001	3.298814777
SMil_00006919	0.240314	2.349642444	0.010872267	3.289448629
SMil_00008799	1.665295	16.24181911	0.017645715	3.285863553
SMil_00007043	1.716088333	16.45678178	0.01743669	3.261486515
SMil_00014043	0.149008667	1.416530111	0.02015677	3.248893121
SMil_00001455	0.431376	4.081313333	0.000234453	3.242015656
SMil_00026489	2.96752	27.14167889	0.047814267	3.193180305
SMil_00027340	1.108134667	9.985433667	0.0362445	3.171691869
SMil_00006651	3.152096667	28.12332222	0.00118373	3.157383346
SMil_00005199	3.06428	26.81434556	0.036490523	3.129385007
SMil_00012730	0.183268333	1.596016667	0.015243782	3.122446286
SMil_00015386	0.136512233	1.171172778	0.014814949	3.100851779
SMil_00026082	6.873713333	58.60788889	0.028403383	3.09193328
SMil_00025350	0.710613	5.964186667	0.005440749	3.069189425
SMil_00001140	0.492215667	4.058209222	0.000745231	3.043480763
SMil_00026819	0.282091333	2.322385222	0.001946321	3.041373049
SMil_00015244	0.298542	2.445548889	0.035975878	3.034152491
SMil_00025745	1.260283667	10.29901889	0.007918027	3.03068651
SMil_00003635	0.097022	0.792228889	0.025111024	3.029533486
SMil_00006366	0.521028	4.223056667	0.02208425	3.018854796
SMil_00021944	0.050411667	0.403588	0.032712377	3.001053722
SMil_00000241	0.476187333	3.804543444	0.000414919	2.998122186
SMil_00016331	0.722215632	5.767783556	0.007681312	2.997515474
SMil_00026640	0.426614333	3.339680222	0.022804568	2.968705627
SMil_00002432	0.391083036	3.05012	0.000757645	2.963319141
SMil_00005002	0.360852	2.807013778	0.011515696	2.959556989
SMil_00002427	0.98748	7.602976667	0.000831038	2.94474093
SMil_00003781	0.218626333	1.674323111	0.031596581	2.937038878
SMil_00000734	11.38841667	85.44508889	0.001091236	2.90743039
SMil_00022011	0.308506667	2.304481222	0.014501116	2.901068441
SMil_00022358	0.388195	2.896756556	0.007988836	2.899585004
SMil_00030266	0.41952	3.110914444	0.04548511	2.890527223
SMil_00022372	0.327272	2.426211778	0.040878279	2.890143403
SMil_00017290	1.075899333	7.869014444	8.79687E-05	2.870639858

SMil_00006534	0.181435	1.314708	0.009394524	2.857217716
SMil_00012827	0.223530667	1.616557778	0.008301904	2.854380395
SMil_00000242	0.420495	3.029785111	0.000649487	2.849054923
SMil_00004938	0.27623	1.980224556	0.0113446	2.841722122
SMil_00007056	0.886806667	6.311042222	0.002041646	2.831186754
SMil_00017382	4.794226667	33.67132222	0.043749436	2.81215035
SMil_00006114	5.43526	38.09885333	0.043381894	2.809326623
SMil_00011192	0.233243667	1.624145333	0.001039174	2.799770922
SMil_00016745	0.194858333	1.347993	0.011047595	2.790315468
SMil_00009238	0.372559333	2.567291111	0.019273414	2.784704785
SMil_00014409	1.676704	11.51002111	0.003011138	2.779190552
SMil_00019502	1.542412	10.58181722	0.028022157	2.778327318
SMil_00014601	0.744736	5.108295556	0.017471067	2.778040996
SMil_00005079	2.560144667	17.52780778	0.005008992	2.775348328
SMil_00009051	1.734512667	11.87258222	0.00014843	2.775031466
SMil_00030124	1.130968667	7.659305556	0.013376948	2.759654633
SMil_00003382	0.220944667	1.489870778	0.013216732	2.753430194
SMil_00013569	0.449488667	3.0058	0.001685376	2.741392373
SMil_00006836	0.353952333	2.356925889	0.017377054	2.735279404
SMil_00001131	0.1200371	0.799010778	0.022486572	2.734734594
SMil_00020526	4.193383333	27.74896667	0.015248182	2.726247425
SMil_00020739	1.788363333	11.82698878	0.015552022	2.725371025
SMil_00019244	4.81626	31.84756667	0.000247535	2.725197965
SMil_00007273	14.56493	95.7402	0.002722966	2.716626052
SMil_00009351	0.208757333	1.369683333	0.039463667	2.713943602
SMil_00012577	1.891976667	12.34260667	0.005383958	2.705680912
SMil_00025006	1.094504667	7.139993333	0.035007008	2.705644621
SMil_00003158	0.631464667	4.111898667	0.049031495	2.703030795
SMil_00004362	155.044	1001.721111	0.004467859	2.691731302
SMil_00027409	1.300897333	8.391775556	0.000462262	2.689468983
SMil_00001077	0.476546	3.065724444	0.023939131	2.685540643
SMil_00009552	0.627563333	4.035688889	2.35246E-05	2.684981994
SMil_00005290	0.701101667	4.506367778	0.026381722	2.684269491
SMil_00027692	0.832294	5.344442222	0.0023228	2.682874246
SMil_00003518	8.924123333	57.09786111	0.041689352	2.677654346
SMil_00002046	0.650894667	4.156402222	0.003822594	2.674839275
SMil_00009183	0.514862667	3.280626667	4.82893E-05	2.671711858
SMil_00007047	0.082300667	0.524206	0.002240688	2.671157844
SMil_00003483	1.706462	10.86712889	0.001484914	2.670890635
SMil_00001722	0.081141	0.512244667	0.004053717	2.658330071
SMil_00000736	0.504014333	3.160374333	0.004372196	2.648558783
SMil_00011950	1.010721	6.311321111	0.001633069	2.642557218
SMil_00018282	1.209191667	7.544954444	0.000840412	2.641469248
SMil_00000952	0.399275	2.490999889	0.006898893	2.641270309
SMil_00020471	1.763904	10.95950111	0.028993182	2.635338177
SMil_00018562	0.330606333	2.047641111	0.004204567	2.63077661
SMil_00008687	0.192714	1.187685111	0.01350297	2.6236191
SMil_00003946	0.48250532	2.939319667	0.010897624	2.606865514
SMil_00001696	0.287463333	1.737329889	0.009582185	2.595421869
SMil_00006097	1.269015	7.665919667	0.027851316	2.594749758
SMil_00012933	0.544613333	3.272104667	0.045700001	2.586914689
SMil_00008116	0.542198	3.252682	0.027774752	2.584738087
SMil_00015442	0.383585	2.296894556	0.031885684	2.582066418
SMil_00021820	11.36970333	68.0491	0.009054668	2.58138147
SMil_00003740	0.081247167	0.485140722	0.032250064	2.578013875
SMil_00004463	1.124142	6.686913333	0.039331221	2.572516133
SMil_00001719	2.699803333	15.97907222	0.000257814	2.565257421

SMil_00014838	0.868426333	5.119371111	0.000565987	2.559491215
SMil_00024514	44.6889	261.2191778	0.011112067	2.547272379
SMil_00012230	0.501890667	2.929436556	0.040381352	2.545178182
SMil_00001065	0.731933333	4.271368889	0.018112946	2.544914344
SMil_00002100	1.266607667	7.276679778	0.023507482	2.522310607
SMil_00013693	2.041363333	11.65946111	0.044922663	2.513896221
SMil_00010511	37.6448	214.1416222	0.019943485	2.508042737
SMil_00014881	15.40383333	87.335	0.047912719	2.503270519
SMil_00011805	0.243800667	1.380552556	0.014021984	2.501471834
SMil_00024761	0.45871	2.596527556	0.027829395	2.500929272
SMil_00025817	10.57937667	59.75573556	0.002757982	2.497822568
SMil_00014228	21.71636667	121.6654778	0.002264794	2.486065212
SMil_00013559	0.472616667	2.641164444	0.006950551	2.482431718
SMil_00029576	8.205066667	45.84731	0.03866448	2.482250128
SMil_00008307	2.662299333	14.64791556	0.003471786	2.459950686
SMil_00029381	0.94824	5.180065556	0.000503619	2.449646199
SMil_00013413	0.081298667	0.442598889	0.023108094	2.444696233
SMil_00016107	1.136496	6.187032222	0.00152981	2.444654941
SMil_00006236	4.01482	21.64628667	0.017851081	2.430712345
SMil_00012751	12.16767333	64.7446	0.037157626	2.411706544
SMil_00015205	1.094686667	5.82222	0.014288255	2.41105137
SMil_00000494	0.276539	1.469492222	0.002162695	2.409762861
SMil_00005242	5.082856667	26.73975111	0.002532237	2.395274584
SMil_00003900	4.290216667	22.44103222	0.003884936	2.387016623
SMil_00015832	0.106305333	0.555600556	0.049101845	2.385834063
SMil_00028576	0.607146	3.16091	0.000165173	2.380224571
SMil_00009435	1.749126667	9.079172222	0.010652193	2.375925999
SMil_00000526	0.273554667	1.410880111	0.027283872	2.366694328
SMil_00006299	1.827246667	9.389793333	5.48874E-05	2.361422003
SMil_00001433	0.219817667	1.124800667	0.00965165	2.35529011
SMil_00008572	2.557853333	13.08663778	0.005172134	2.355089039
SMil_00021328	0.477589	2.439582889	0.005650707	2.352792989
SMil_00011506	4.496683333	22.91277333	0.005484108	2.349218896
SMil_00004013	2.202734667	11.19164	0.046301791	2.345053833
SMil_00001193	1.636250667	8.311624444	0.001008276	2.344736688
SMil_00000149	0.213340333	1.082206111	0.012701786	2.342746646
SMil_00016647	0.537364	2.725661111	0.015935447	2.342634621
SMil_00016214	0.391857667	1.985623333	0.002282323	2.341190345
SMil_00020713	0.337887667	1.710982111	0.020918642	2.34020908
SMil_00009035	8.18663	41.35547778	0.001232544	2.336736837
SMil_00004650	5.844073333	29.50592778	0.011592103	2.335958636
SMil_00006585	7.279243333	36.63196667	0.00034351	2.33124276
SMil_00025418	1.540047667	7.733157778	0.017584981	2.328082643
SMil_00005499	0.442576667	2.222043333	0.012798933	2.327887655
SMil_00008706	1.137868333	5.708732222	0.000397746	2.326836764
SMil_00007861	1.77396	8.875223333	0.000959486	2.322809944
SMil_00000284	0.719576333	3.58686	0.002167617	2.317501793
SMil_00029845	0.468871667	2.334818333	0.036238046	2.316045295
SMil_00019935	0.518097667	2.570673556	0.042859425	2.310850426
SMil_00021892	2.355126667	11.67645333	0.000126982	2.309725569
SMil_00005254	0.368386	1.824266222	0.01786755	2.308026143
SMil_00028131	1.534627667	7.596514444	0.033977225	2.30744894
SMil_00023884	3.707146667	18.33570111	0.005724801	2.306274333
SMil_00014915	10.48127333	51.78349444	0.01408675	2.304678329
SMil_00023882	1.47173	7.26494	0.004126743	2.303437861
SMil_00021138	0.525917	2.594589	0.0375554	2.302598987
SMil_00002320	9.983446667	49.13014444	0.004036984	2.298998601

SMil_00004983	55.87913333	274.0835556	0.013913827	2.294234222
SMil_00015477	4.166343333	20.41421111	0.000675629	2.29272018
SMil_00006406	1.236858667	6.054413333	0.002033773	2.291306515
SMil_00012005	28.9994	141.6637333	0.025342331	2.288375512
SMil_00004962	0.920039	4.479519333	0.005668051	2.283577012
SMil_00011390	0.146518333	0.713108	0.009557616	2.283039394
SMil_00007690	2.045598333	9.911198889	0.013493028	2.276536691
SMil_00005030	0.663063333	3.212215556	0.019522651	2.276350125
SMil_00013928	4.964916667	23.92746	0.036979432	2.268825849
SMil_00025673	0.447736667	2.156308667	0.003049107	2.267841333
SMil_00006528	0.850245667	4.092743333	0.00826355	2.267116541
SMil_00026330	1.93866	9.330772222	0.002619144	2.266936677
SMil_00026490	32.081	154.3844222	0.019829358	2.26673617
SMil_00027908	0.256363667	1.228189889	0.026256935	2.260269917
SMil_00007219	1.316332333	6.293506667	0.000541761	2.257340322
SMil_00002904	0.341213667	1.630505444	0.007268047	2.256571921
SMil_00026555	0.537195333	2.564391111	0.005739619	2.255097636
SMil_00023377	2.30964	10.98401778	0.000358024	2.249665962
SMil_00014073	0.983446	4.676372222	0.001756407	2.249472025
SMil_00029442	0.662041667	3.129156667	0.008763255	2.240779968
SMil_00011863	3.293594333	15.55474778	0.028688032	2.239620225
SMil_00019622	1.403323	6.610758889	0.019804791	2.235968788
SMil_00015984	3.716366667	17.46213111	0.000929667	2.232264885
SMil_00012278	0.857303667	4.022966444	0.01107721	2.230381486
SMil_00022421	1.719064333	8.041927778	0.007557485	2.225917843
SMil_00029150	17.66673333	82.41557778	0.004127751	2.221881751
SMil_00029281	1.060373333	4.944687778	0.026184161	2.221307133
SMil_00029633	0.1453096	0.677303222	0.017193737	2.22067184
SMil_00007769	0.396124333	1.842527222	0.039148886	2.217660703
SMil_00003681	0.987193	4.588922444	0.020573371	2.216751355
SMil_00008560	1.368078667	6.323926667	0.001940613	2.208669448
SMil_00023522	28.80163333	133.0527111	0.029608294	2.207775373
SMil_00008015	3.01301	13.91463778	0.024949324	2.207325987
SMil_00017439	2.082096667	9.614753333	0.001602329	2.207212795
SMil_00010974	0.391405333	1.806379	0.0052184	2.2063653
SMil_00008752	5.64167	26.03003	0.002987316	2.205982789
SMil_00002916	1.80565	8.326391111	0.016349049	2.205173053
SMil_00006709	0.427853	1.968587778	0.026664616	2.201973931
SMil_00024895	6.464983333	29.67167111	0.031716002	2.198367626
SMil_00025219	0.520819	2.389969556	0.00787182	2.198138255
SMil_00002155	0.164493667	0.751629889	0.026122801	2.191990399
SMil_00018115	0.334015667	1.522723889	0.005404218	2.188666688
SMil_00018144	1.251953333	5.702751111	0.005230212	2.187477283
SMil_00004094	0.935943	4.258666667	0.000938	2.185909236
SMil_00019732	0.473827667	2.153487333	0.021818481	2.184240493
SMil_00009584	1.070674	4.843315556	0.002596565	2.177475728
SMil_00007385	20.94020667	93.61298889	0.002795914	2.160433038
SMil_00000014	1.838890667	8.206562222	0.008056546	2.15794229
SMil_00014947	2.952596667	13.17063556	0.000890473	2.157268767
SMil_00022164	1.230090667	5.485265444	0.047149287	2.156796781
SMil_00025997	0.538252667	2.397051667	0.033268454	2.154905538
SMil_00007097	2.13678	9.483255556	0.023247233	2.149944037
SMil_00006856	0.612429667	2.717727444	0.012355362	2.149784703
SMil_00022353	2.127296667	9.401632222	0.006547175	2.143890004
SMil_00026737	1.81995	8.03852	0.002169597	2.143031091
SMil_00009171	108.5777333	479.3836667	0.007942214	2.142452483
SMil_00027808	1.481677333	6.536873222	0.008905383	2.141369413

SMil_00000062	12.6331	55.60218889	0.047650151	2.137932977
SMil_00006095	3.942636667	17.33619222	0.00821658	2.136554385
SMil_00011316	0.328693667	1.443868	0.003873031	2.135123292
SMil_00011056	4.657256667	20.43772444	0.014655697	2.133682276
SMil_00013467	0.234564333	1.024837556	0.048052826	2.127339684
SMil_00017903	1.161212667	5.019887778	0.000407162	2.112022898
SMil_00014271	0.712786333	3.078578889	0.000943982	2.110722958
SMil_00002672	13.08317	56.38154444	0.00909901	2.107510855
SMil_00004641	1.89676	8.153633333	0.00182617	2.103905937
SMil_00024978	0.422857333	1.812164	0.002351165	2.099470621
SMil_00000656	1.458020667	6.237165556	0.001184655	2.096879384
SMil_00019462	1.550799333	6.632422222	0.000478288	2.096523832
SMil_00012329	1.51177	6.436425556	0.009149088	2.09002105
SMil_00029543	0.183059	0.778548222	0.016869496	2.088477696
SMil_00009232	2.791036667	11.85937444	0.025459612	2.08715493
SMil_00019024	0.240353667	1.019952556	0.038093603	2.085271327
SMil_00002941	0.200734667	0.850973556	0.046400894	2.08382451
SMil_00000932	0.675721667	2.86086	0.001150151	2.081947879
SMil_00018558	2.108911	8.908752222	0.006690879	2.078725167
SMil_00019679	0.461620333	1.949735556	0.008884246	2.078499785
SMil_00015098	1.537515667	6.476134444	0.001494453	2.074531825
SMil_00012745	1.010286333	4.254845556	0.031493037	2.074342529
SMil_00015393	5.052343333	21.20125556	0.000913545	2.069125119
SMil_00010989	2.079656667	8.723212222	0.003229732	2.068514117
SMil_00025406	3.005341333	12.59248556	0.000476014	2.066962316
SMil_00005674	1.522155667	6.369401111	0.00550784	2.065041821
SMil_00018221	1.991433333	8.30643	0.047566536	2.060421374
SMil_00029482	1.213675	5.052933222	0.014475879	2.057738968
SMil_00012939	1.593193333	6.626701111	0.026509278	2.056369502
SMil_00021389	20.1669	83.67481111	0.006390275	2.052804055
SMil_00014457	0.746178	3.085660444	0.035230506	2.047987582
SMil_00016994	6.412293333	26.47391111	0.000890923	2.045659018
SMil_00025452	5.939216667	24.50951556	0.023415766	2.0449974
SMil_00029753	0.549064667	2.265513889	0.021233028	2.044790356
SMil_00001525	1.05103	4.33573	0.039704939	2.044471069
SMil_00002459	0.827571	3.411208889	0.000635351	2.043328107
SMil_00010685	0.275893333	1.136167222	0.000355923	2.041992687
SMil_00014342	0.436874	1.797619	0.018772997	2.040798125
SMil_00020522	0.375572333	1.542069444	0.045443566	2.037705041
SMil_00000852	2.287655	9.328037778	0.040822783	2.027704135
SMil_00012728	1.472698333	5.976632222	0.000290948	2.020870828
SMil_00029198	0.916676667	3.715917778	0.016810782	2.019233723
SMil_00017100	0.523735667	2.122442556	0.031223181	2.018814746
SMil_00022716	16.78384667	67.95964444	0.004245702	2.017604902
SMil_00000556	0.270863667	1.096194	0.004439241	2.016864353
SMil_00030289	0.759936333	3.074977778	0.001543511	2.016625523
SMil_00016533	9.662203333	38.75982667	0.031388686	2.004138
SMil_00003340	1.243146667	4.984891	0.033567506	2.003565443
SMil_00006118	2.403923333	9.619966667	0.002613646	2.000641009

Table S4 Amino acid Sequences used in the phylogenetic analysis

SmMYB97

MGRAPCCEKVGLRRGRWTAEEDEKLTKYIEENGEGSWRSLPKNAGLLRCGKSCRLRWINYLRSDV
KRGNISAEEEEIIINLHASKGNRWSLIAAHLPGRTDNEIKNYWNSHLSRKIHSFRRPNPNFIPPPPSSKK
ATSVNRRGSRKRPGHQEDAAVVMPTTPTPEREAAGRIATEERESGSSISMLGDIVEDLSGLWGPEFEI
SRVGPGSENGDTGETSFKCNEFGLGETGSGLGVSSEIYENTFSWMLDDDCDEIWDSTHQGLDDVMFS
WLLS

SmMYB6

MGRSACCSKVGLRRGPWSTKEDSLLASYIQQHGEGQWRSLPKKAGLLRCGKSCRLRWMNYLRPGI
KRGNISEDEEDLIVRLHRLLGNRWSLIAGRLPGRTDNEIKNYWNTHLLKKLNTAAAAADKKKIKKPP
KKSAAAAAASQDMKNSKVYAPKPMRVSSRSDSDDSLGSSDGAPEVLVSAWPEVEDPAALYHFSDS
FADDMWEKVYAEYMQLL

SmMYB111

MGRSPCCSKVGLRRGPWSTKEDSLLANYIQQNGEGQWRSLPKKAGLLRCGKSCRLRWMNYLRPGI
KRGNISEDEEDLIVRLHGLLGNRWSLIAGRLPGRTDNEIKNYWNTHLLKKLKTAAAPHKDLPNLAAK
PKKKKPKQKPTPPSPLKDESAADEPTPPPKTKVYLPKPIRVSSAFSRSNSYDSLASNSDGEKAAEELSY
VPLQWPPIFELEEGDYGVCAAVGGGSDDFLDGGFILPVLNHSDSISSDVNMLEKVYDEYLQLL*

SmMYB112

MVASTSIKEKQRPSKKRGPWTAEEDQEITAAVAVHGAKQWATIAASSGLSRGAKSCRVRWMNYLR
PNIMSDQEEDLIIRLHKLLGNRWSLIAARLPGRTDNEIKNYWKHHLSKKRLEKGVVVAGISSDQHKG
TSVSSKSVEISVSEAKIDDFFDFSNEHPSTLEWVTKFLEFGDT

ZmC1

MGRRACCAKEGVKRGAWTSKEDDALAAYVKAHGEGKWREVPQKAGLRRCGKSCRLRWLNYLRP
NIRRGNISYDEEDLMIIRLHRLLGNRWSLIAGRLPGRTDNEIKNYWNSTLGRRAGAGAGAGGSRVVIA
PDTGSHATPAATSGSGETGQKGAAPRADPDSAGTTTTSAAAVWAPKAVRCTGGLFFFHRDTTPAHA
GETATPMAGGGLGGEAGSSEDCSSAASVSPLVGSQDEPCFSGDGDCDWMDDVRALASFLESDEDW
LRCQTAGQLA

AtMYB5

MMSCGGKKPVSKKTTPCCTKMGMKRGPWTVEEDEILVSFIKKEGEGRWRSLPKRAGLLRCGKSCR
LRWMNYLRPSVKRGGITSDEEDLILRLHRLLGNRWSLIAGRIPGRTDNEIKNYWNTHLRKKLLRQGI
DPQTHKPLDANNIHKPEEEVSGGQKYPLEPISSSHTDDTTVNGGDGDSKNSINVFGGEHGYEDFGFC
YDDKFSSFLNSLINDVGDPFGNIIPISQPLQMDDCKDGIVGASSSSLGHD

AtMYB12

MGRAPCCEKVGIKRGRWTAEEDQILSNYIQSNGEGSWRSLPKNAGLKRCGKSCRLRWINYLRSDLK
RGNITPEEEELVVKLHSTLGNRWSLIAGHLPGRTDNEIKNYWNSHLSRKLHNFIRKPSISQDVSAVIM
TNASSAPPPPQAKRRLGRTSRSAMKPKIHRTKTRKTKKTSAPPEPNADVAGADKEALMVESSGAEA
ELGRPCDYYGDDCNKNLMSINGDNGVLTFDDDIIDLLLDESDPGHLYTNTTCGGDGELHNIRDSEGA
RGFSDTWNQGNLDCLLQSCPSVESFLNYDHQVNDASTDEFIDWDCVWQEGSDNNLWHEKENPDSM
VSWLLDGDDEATIGNSNCENFGEPLDHDDESALVAWLLS

AtMYB11

MGRAPCCEKVGIKKGRWTAEEDRTLSDYIQSNGEGSWRSLPKNAGLKRCGKSCRLRWINYLRSDIK
RGNITPEEEDVIVKLHSTLGTRWSTIASNLPGRTDNEIKNYWNSHLSRKLHGYFRKPTVANTVENAPP
PPKRRPGRTSRSAMKPKFILNPKNHKTPNSFKANKSDIVLPTTTIENGEGDKEDALMVLSSSSLSGAE
EPGLGPCGYGDDGDCNPSINGDDGALCLNDDIFDSCFLLDDSHAVHVSSCESNNVKNSEPYGGMSV
GHKNIETMADDFVDWDFVWREGQTLWDEKEDLDSVLSRLLDGEEMESEIRQRDSNDFGEPLDIDEE
NKMAAWLLS

AtMYB111

MGRAPCCEKIGLKRGRWTAEEDEILTKYIQTNGEGSWRSLPKKAGLLRCGKSCRLRWINYLRRDLK
RGNITSDEEEIIVKLHSLLGNRWSLIATHLPGRTDNEIKNYWNSHLSRKIYAFTAVSGDGHNLLVNDV
VLKKSCSSSSGAKNNNKTKKKKKGRTSRSSMKKHKQMVTASQCFSQPKELESDFSEGGQNGNFEGE
SLGPYEWLDGELERLLSSCVWECTSEEAVIGVNDEKVCESGDNSSCCVNLFEEEQGSETKIGHVGITE
VDHDMTVEREREGSFLSSNSNENNDKDWWVGLCNSSEVGFGVDEELLDWEFQGNVTCQSDDLWD
LSDIGEITLE

AtMYB123

MGKRATTSVRREELNRGAWTDHEDKILRDYITTHGEGKWSTLPNQAGLKRCGKSCRLRWKNYLRP
GIKRGNISSDEEELIIRLHNLLGNRWSLIAGRLPGRTDNEIKNHWNSNLRKRLPKTQTKQPKRIKHSTN
NENNVCVIRTKAIRCSKTLLFSDLSLQKKSSTSPLPLKEQEMDQGGSSLMGDLEFDFDRIHSEFHFPDL
MDFDGLDCGNVTSLVSSNEILGELVPAQGNLDLNRPFTSCHHRGDDEDWLRDFTC

AtPAP1

MEGSSKGLRKGAWTTEEDSLLRQCINKYGEGKWHQVPVRAGLNRCRKSCRLRWLNYLKPSIKRGK
LSSDEVDLLLRLHRLLGNRWSLIAGRLPGRTANDVKNYWNTHLSKKHEPCCKIKMKKRDITPIPTTP
ALKNNVYKPRPRSFTVNNDCNHLNAPPKVDVNPPCLGLNINNVCDNSIIYNKDKKKDQLVNNLIDG
DNMWLEKFLEESQEVDILVPEATTTEKGDTLAFDVDQLWSLFDGETVKFD

AtPAP2

MEGSSKGLRKGAWTAEEDSLLRLCIDKYGEGKWHQVPLRAGLNRCRKSCRLRWLNYLKPSIKRGR
LSNDEVDLLLRLHKLLGNRWSLIAGRLPGRTANDVKNYWNTHLSKKHESSCCKSKMKKKNIISPPTT
PVQKIGVFKPRPRSFSVNNGCSHLNGLPEVDLIPSCLGLKKNNVCENSITCNKDDEKDDFVNNLMNG
DNMWLENLLGENQEADAIVPEATTAEHGATLAFDVEQLWSLFDGETVELD

AtPAP4

MEGSSKGLRKGAWTAEEDSLLRQCIGKYGEGKWHQVPLRAGLNRCRKSCRLRWLNYLKPSIKRGK
FSSDEVDLLLRLHKLLGNRWSLIAGRLPGRTANDVKNYWNTHLSKKHEPCCKTKIKRINIITPPNTPA
QKVDIF

MdMYB22

MGRAPCCEKVGLKKGRWTAEEDEILLNYIQANGEGSWRSLPKNAGLLRCGKSCRLRWINYLRADL
KRGNISSQEEDIIIKLHASLGNRWSLIASQLPGRTDNEIKNYWNSHLSRKIGTFRRPATTTVITTEISTSV
PPAGDEVSAAMELGPPKRRGGRTSRWAMKKNKTYSTTKPKGLKARLRQSHHKHDNIAAAAADDD
RLNNAHEAIALPTKSNNKNVDTMQDGYVLLMEVPDQQQETRGGGIAMPATVIDHQKETDGEKLILG
PHPHGHDDDDMNEYVGINGGLLGFTDYLMDDINEDEILDPNGVMALSSSEINIHQDADHFPDAVISD
HQDTELPSSCDQLVICPNKVMTTTTTYYGIQ

MdMYB12

MGRSPCCAKEGLNRGAWTAHEDKVLTQYIKLHGEGRWRNLPKKAGLKRCGKSCRLRWLNYLRPDI
KRGNISPDEEELIIRLHKLLGNRWSLIAGRLPGRTDNEIKNYWNTNLGKKVPDRQQQRSASNLKHHK
NGEPNSKKAKSMDMASPSSLVYRTKAVKCTQVFINPQPHKVLLGHDHQHCTEETNTVLMFDGKPA
AMDDDHINRTLSFSSFSNINADQENSTSDFLVDFDMNEISIASLLNSDFPEINRDYLNHNNSDTITSHFV
DETAQFFSEEMLQHWNNGGSDDGDNQVQVQPNLALNFHSFTSFLGSDHQGEEWLGVGESS

MdMYB10

MEGYNENLSVRKGAWTREEDNLLRQCVEIHGEGKWNQVSYKAGLNRCRKSCRQRWLNYLKPNIK
RGDFKEDEVDLIIRLHRLLGNRWSLIARRLPGRTANAVKNYWNTRLRIDSRMKTVKNKSQEMRKTN
VIRPQPQKFNRSSYYLSSKEPILDHIQSAEDLSTPPQTSSSTKNGNDWWETLLEGEDTFERAAYPSIEL
EEELFTSFWFDDRLSPRSCANFPEGQSRSEFSFSTDLWNHSKEE

VvMYBA1

MESLGVRKGAWIQEEDVLLRKCIEKYGEGKWHLVPLRAGLNRCRKSCRLRWLNYLKPDIKRGEFAL
DEVDLMIRLHNLLGNRWSLIAGRLPGRTANDVKNYWHSHHFKKEVQFQEEGRDKPQTHSKTKAIKP
HPHKFSKALPRFELKTTAVDTFDTQVSTSRKPSSTSPQPNDDIIWWESLLAEHAQMDQETDFSASGE
MLIASLRTEETATQKKGPMDGMIEQIQGGEGDFPFDVGFWDTPNTQVNHLI

VvMYBA2

MKSLGVRKGAWTQEEDVLLRKCIEKYGEGKWHLVPLRAGLNRCLKSCRLRWLNYLKPDIKRGEFA
LDEVDLMIRLHNLLGNRWSLIAGRLPGRTANDVKNYWHGHHLKKKVQFQEEGRKKPQTHSKTKAI
KPHPHKFSKALPRFELKTTAVDTFDTQVSTSSKPSSTSPQPNDDIIWWESLLAEHAQMDQETDFSASG
EMLIASLWTEETATQKKGTHSKTKAIKPHPHKFSKALPRFELKTTAVDTFDTQVSTSSKLIHVTTTE

VvMYBPA1

MGRAPCCSKVGLHRGSWTAREDTLLTKYIQAHGEGHWRSLPKKAGLLRCGKSCRLRWMNYLRPDI
KRGNITPDEDDLIIRLHSLLGNRWSLIAGRLPGRTDNEIKNYWNTHLSKKLRSQGTDPNTHKKMTEPP
EPKRRKNTRTRTNNGGGSKRVKISKDQENSNHKVHLPKPVRVTSLISMSRNNSFESNTVSGGSGSSS
GGNGETLPWPSFRDIRDDKVIGVDGVDFFIGDDQGQDLVASSDPESQSHMPPTDNSLEKLYEEYLQL
LEREDTQVQLDSFAESLLI

VvMYBPA2

MGRRPCCAKEGLNRGSWSAWEDKILCNYVEVHGEGKWRDLPQRAGLKRCGKSCRLRWLNYLRPD
IKRGNISSEEEELIIRLHKLLGNRWSLIAGRLPGRTDNEIKNYWNTNLSKRLQASKGQNSPNKKVENP
KNQTSGTGKSSAELHTVIRTRAVRCSKVIIPRVQADFDENPSPKMAVPTSEPSSSALEQGETANFFMG
FDIGDLLTSDALNSFLDQDEEMGENNSNGVSDHFPPCSDFLAPEIENQEGVSGLLQPSEALELKTLASF
LNSEDEWITENNQVP

ZmP1
MGRAPCCEKVGLKRGRWTAEEDQLLANYIAEHGEGSWRSLPKNAGLLRCGKSCRLRWINYLRADV
KRGNISKEEEDIIIKLHATLGNRWSLIASHLPGRTDNEIKNYWNSHLSRQIHTYRRKYTAGPDDTAIAI
DMSKLQSADRRRGGRTPGRPPKASASRTKQADADQPGGEAKGPAAAASSPRHSDVVNPGPNQPNSS
SGSTGTAEEEGPSSEDASGPWVLEPIELGDLVWGEADSEMDALMPIGPGGHDSAALEGLGAVGCEA
QVDDLFDMDWDGFAAHLWGGPEQDEHSAQLRQAAEPLEVAAAAAAATAARTPDDRELEAFETWL
LSDSF

ZmP2

MGRAPCCEKVGLKRGRWTAEEDQLLANYIAEHGEGSWRSLPKNAGLLRCGKSCRLRWINYLRADV
KRGNISKEEEDIIIKLHATLGNRWSLIASHLPGRTDNEIKNYWNSHLSRQIHTYRRKYTAGPDDTAIAI
DMSKLQSADRRRGGRTPGRPPKTSASRTKHSDADQPGGEAKGAAASSPRHSDAVNPGPNQPNSSSG
STGTAEEEGPSSEDASGPWVLEPIELGDLWGEADSEMDALMPIGPGGHDSAALQGLGAVGGEAQVD
DLFDMDWDGFAAHLWGGPEQDDHSAQLRQAAEPMEAAAVAAAAAAATAACTPDDRELEAFETW
LLSDSF

ZmMYB148

MGKGRAPCCAKVGLNKGSWTPEEDMRLIAYIQKYGHANWRALPKQAGLLRCGKSCRLRWINYLRP
DLKRGNFTAEEEEAIIKLHGLLGNKWSKIASCLPGRTDNEIKNVWNTHLKKRVSPAGEERGAAGSKK
KKKKKTTKAAAGGGAEAPLPLPSPSPSSSTTTTNFSSGDSGEQQSNNMSKEADDELDLENFEMMPM
LDVDDPSFGFGTLVDTAPAPYGSAVSVSASAATSPCASSTSPPPASAPPGVDDLLVLPEIDMGHELWS
IIDGDAAEAPAPRCQRNPAEPTNGADAGSHGAEGKEWWLEDLERELGLWGTVEDYQYPMGPQGLL
VADHPDPLPAMVDDPVSCYFQAGPASAVLQELPGYPVPATAVTGSINQMGL

ZmMYB111

MGRQPCCDKLGVKRGPWTAEEDRKLINFILTNGHCCWRAVPKLAGLLRCGKSCRLRWTNYLRPDL
KRGLLTDAEEQVVIDLHAKLGNRWSKIAAKLPGRTDNEIKNHWNTHIKKKLIKMGIDPVTHEPLDRK
TTSSGPATTSQSTKSDEATKEQSPQNDDAVIRDVPADGCSPTESSTNTVSTGGSSSSGGGGHDQDPLV
KWLLEEEPATGDEAWLNFTGSVDVDEFSSIAAGPELLPWDGATDWLLDYQDFGLGDSSLVDGYMV
NNNSSNGAKF
